# Uniform regulation of stomatal closure across temperate tree species to sustain nocturnal turgor and growth

**DOI:** 10.1038/s41477-025-01957-3

**Published:** 2025-04-03

**Authors:** Richard L. Peters, Matthias Arend, Cedric Zahnd, Günter Hoch, Stefan K. Arndt, Lucas A. Cernusak, Rafael Poyatos, Tobias Zhorzel, Ansgar Kahmen

**Affiliations:** 1 Department of Environmental Sciences—Botany, University of Basel, Basel, Switzerland; 2 Tree Growth and Wood Physiology, TUM School of Life Sciences, Technical University of Munich, Freising, Germany; 3 Department of Environmental Sciences, Plant Ecology, University of Trier, Trier, Germany; 4 School of Biological Sciences, University of Utah, Salt Lake City, UT USA; 5 School of Agriculture, Food and Ecosystem Sciences, The University of Melbourne, Richmond, Victoria Australia; 6 College of Science and Engineering, James Cook University, Cairns, Queensland Australia; 7 CREAF, Bellaterra (Cerdanyola del Vallès), Spain; 8 Universitat Autònoma de Barcelona, Bellaterra (Cerdanyola del Vallès), Spain

**Keywords:** Plant physiology, Ecophysiology, Forest ecology

## Abstract

Water loss and carbon gain are balanced by stomatal control^1^, a trade-off that has allowed trees to survive and thrive under fluctuating environmental conditions^2–4^. During periods of lower water availability, stomatal closure prevents excess water loss^5^. Various strategies of stomatal control have been found among tree species^6,7^, but the trigger for this behaviour remains elusive. We found a uniform pre-dawn water potential threshold (−1.2 MPa) for stomatal closure across species, which coincided with stem-growth cessation. Meanwhile, midday water potentials at stomatal closure were more variable across species and stomatal control did not follow species-specific thresholds of hydraulic failure, a commonly adopted theory in plant biology^8–10^, and often used in predictive water-use modelling^11,12^. This indicates that nocturnal rehydration, rather than daytime hydraulic safety is an optimization priority for stomatal closure in trees^13^. We suggest that these processes are critical for forecasting the global carbon cycle dynamics.

## Abstract (web summary)

Stomatal closure in trees occurs at a pre-dawn water potential of −1.2 MPa, aligning with growth cessation. This suggests that nocturnal rehydration, not hydraulic safety, drives stomatal control, with implications for tree water use and carbon cycling.

## Main

Plants must balance water loss through stomatal pores on the leaf surface with carbon uptake via photosynthesis. In the short term—daily or seasonally—plants can reduce water loss to the atmosphere by reducing stomatal conductance (*g*_s_) to water vapour. While stomatal closure decreases transpiration during periods of low water availability, it also constrains carbon uptake^1^. Understanding the environmental and physiological thresholds for stomatal closure in mature trees is crucial, as forests are estimated to absorb around a fourth of the annual anthropogenic carbon emissions^14^. However, different tree species have been reported to reduce *g*_s_ at varying environmental thresholds, creating uncertainty in predictions of global water and carbon cycles^6^. Explaining *g*_s_ responses to low water availability is of particular importance, as global observations indicate significant variability in *g*_s_ and photosynthesis responses to environmental variables^3,7,15^.

Stomatal closure occurs when adjacent guard cells lose turgor either through passive diffusion of water or via active metabolic pathways^16^. This process responds to a decrease in the internal water status of tissues, typically quantified with leaf water potential measurements (*Ψ*_leaf_)^5^. As *Ψ*_leaf_ decreases, *g*_s_ also decreases. A common explanation for species-specific *g*_s_ responses to midday *Ψ*_leaf_ is that trees optimize carbon uptake against the risk of lethal hydraulic conductance loss in the xylem which occurs at low *Ψ*_leaf_ (refs. ^2,9,17^), known as the ‘safety-efficiency trade-off’. Earth system models use this principle^12^, and recent studies supported this theorem in juvenile trees^8^. These models assume that species-specific stomatal sensitivity to reduced water availability is guided by the species-specific point of loss of plant hydraulic conductivity^4^. However, recent observations in mature trees challenge this trade-off and suggest that maintaining turgor within growing tissues during night-time is the optimization priority for *g*_s_ across temperate tree species^13,18^. Tree growth occurs mainly at night^19^ and depends on the nocturnal water status. Delayed stem rehydration after a day of transpiration due to low water availability in the soil can hamper cambial turgor recovery^20^. Conversely, very negative *Ψ*_leaf_ values, which cause lethal embolisms^10^, typically occur around midday. To test whether trees optimize *g*_s_ primarily to avoid daytime embolisms or are more conservative and induce stomatal closure already around the point of night-time cambial turgor loss, we can compare the uniformity of pre-dawn and midday *Ψ*_leaf_ thresholds for stomatal closure across species. Yet, empirical evidence for such a test is lacking as long-term in situ measurements on tall trees are rare, limiting our general understanding of species-specific *g*_s_ responses to diel *Ψ*_leaf_ dynamics.

**Fig. 1:**
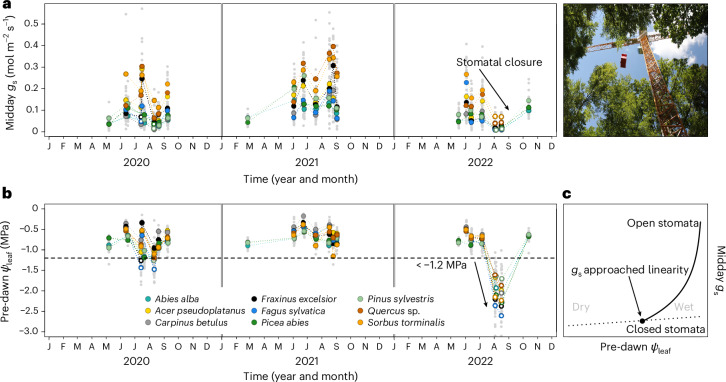
Time series of stomatal conductance (*g*_s_) and pre-dawn leaf water potentials (*Ψ*_leaf_) collected using a canopy crane at the Swiss Canopy Crane II (SCCII) site. **a**, Mean species-specific midday *g*_s_ measured on leaves from the top of the canopy, shown with large, coloured circles. Open circles indicate measurement dates where *Ψ*_leaf_ was below −1.2 MPa. Grey dots represent raw measurements. **b**, Pre-dawn (04:00–06:00 CET) *Ψ*_leaf_, with coloured dots indicating the species mean. In 2022 an exceptional drought caused all *Ψ*_leaf_ to drop below −1.2 MPa (shown with open circles). **c**, Theoretical example of how the point of stomatal closure (*P*_st_) was defined. *P*_st_ was assumed when the negative exponential behaviour of *g*_s_ changed to a linear decrease (Supplementary Methods). Midday *Ψ*_leaf_ values are presented in Supplementary Fig. 3. J through D, months of January through December. Source data

We collected unique empirical evidence to characterize the water status conditions under which mature tall trees (~20–35 m in height and 80–150 years of age; Supplementary Tables 1 and 2) of various species reduce *g*_s_, considering diel *Ψ*_leaf_ dynamics and growth (Fig. 1). Over 3 years of intensive canopy and stem monitoring of 95 trees from 9 common temperate tree species, we made observations across a wide range of environmental conditions (Supplementary Fig. 1). This natural climatic variability allowed us to cover a unique hydration spectrum in the target species. The hydration status of the trees ranged from well hydrated, early in the season (pre-dawn *Ψ*_leaf_ −0.22 to −0.69 MPa; Fig. 1b and Supplementary Table 3), to pre-dawn *Ψ*_leaf_ values below −1.5 MPa (Supplementary Table 3), close to the expected turgor loss points of leaves for these species (Supplementary Table 4). We observed a wide range of *g*_s_ values, from fully open stomata to stomatal closure (Fig. 1a). Using these data, we tested whether stomatal closure is better explained by pre-dawn or midday *Ψ*_leaf_ conditions and if stomatal closure occurs under more uniform pre-dawn or midday *Ψ*_leaf_ conditions across species. We defined the point of stomatal closure (*P*_st_) by analysing the relationship between stomatal conductance (*g*_s_) and leaf water potential (*Ψ*_leaf_). The *P*_st_ was identified as the *Ψ*_leaf_ point where *g*_s_ transitions from a negative exponential decrease to low values^21^ that followed a slight linear decline^10^ (Fig. 1c)^22^.

**Fig. 2:**
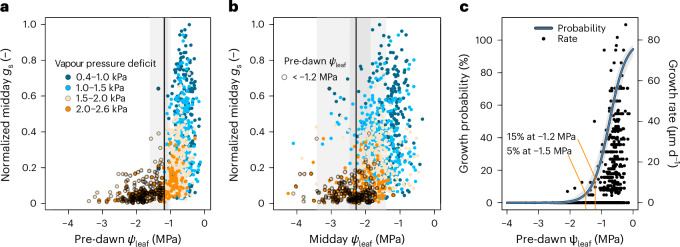
Relationship between pre-dawn and midday *Ψ*_leaf_ and normalized midday *g*_s_ or growth of mature temperate tree species. **a**, The relationship between normalized midday *g*_s_ and pre-dawn *Ψ*_leaf_ for all studied species at the SCCII site. The point of stomatal closure (*P*_st_) was quantified for each species individually. The across-species mean *P*_st_ is indicated by a bold line, with the 50th and 95th percentile ranges shown as grey shading (Supplementary Fig. 4). **b**, Instantaneous response conditions between midday *g*_s_ and midday *Ψ*_leaf_, presented as in **a**. The across-species mean, as well as the 50th and 95th percentile ranges for *P*_st_, are depicted as for **a**. **c**, Growth probability and rate responses to pre-dawn *Ψ*_leaf_, obtained from weekly band dendrometer readings. Zero growth rates versus non-zero growth rates were analysed to determine the growth probability using a mixed-effect model with a binomial distribution. Axes labelled (-) indicate unitless values, resulting from normalization to the maximum value. The grey shading around the line represents the 95% confidence interval. Source data

We first examined how well pre-dawn and midday *Ψ*_leaf_ values can explain the in situ *g*_s_ measurements (Fig. 2). The pre-dawn *Ψ*_leaf_ showed a high explanatory power for the *g*_s_ response across species (*R*^2^ = 0.58, *P* < 0.01), which was greater than that of midday *Ψ*_leaf_ (*R*^2^ = 0.41, *P* < 0.01). As pre-dawn *Ψ*_leaf_ largely reflects soil water availability dynamics, the strong relationship between pre-dawn *Ψ*_leaf_ and *g*_s_ suggests that stomatal responses are influenced by insufficient tissue rehydration caused by drying soils, with stomata remaining closed unless the soil is adequately wet. Thus, pre-dawn *Ψ*_leaf_ appears to be a primary control mechanism for stomatal closure in temperate trees. Including vapour pressure deficit (VPD) in the analysis substantially increased the goodness-of-fit, particularly under wetter conditions with less-negative pre-dawn *Ψ*_leaf_ (*R*^2^ = 0.68, *P* < 0.01 in Supplementary Table 5). This suggests a secondary control mechanism or an association between pre-dawn *Ψ*_leaf_ and the hidden variable controlling stomatal conductance (for example, turgor in the guard cells).

We quantified the pre-dawn and midday *P*_st_ thresholds across species. For these thresholds, we both determined the point where *g*_s_ response to *Ψ*_leaf_ approaches linearity (Fig. 1c) and the point where *g*_s_ crosses a fixed lower *g*_s_ threshold (Supplementary Fig. 4), to confirm the robustness of our *P*_st_ quantification. Importantly, the variance in the estimated point of stomatal closure (*P*_st_) across different species was much smaller for pre-dawn than midday *Ψ*_leaf_ (s.d. of 0.2 versus 0.7 MPa, respectively; Fig. 2). This indicates not only that pre-dawn *Ψ*_leaf_ is a primary control for stomatal closure in temperate trees, but also that a uniform pre-dawn *Ψ*_leaf_ threshold for stomatal closure of approximately −1.2 MPa across species exists.

Our data shed light on several basic aspects of tree water relations and their control mechanisms. *P*_st_ occurred at substantially more negative *Ψ*_leaf_ when considering midday *Ψ*_leaf_ (−2.3 ± 0.7 MPa, Fig. 2b) compared to pre-dawn *Ψ*_leaf_ (1.2 ± 0.2 MPa, Fig. 2a), indicating that if the soil is sufficiently wet, trees allow more negative midday water potentials. Moreover, *P*_st_ coincided with the inflection point of the nonlinear relationship between pre-dawn and midday *Ψ*_leaf_ (ref. ^23^) (referred to as the hydroscape), indicating an important hydraulic threshold at which we defined the *P*_st_ (ref. ^22^) (Supplementary Fig. 5). Furthermore, although it is commonly assumed that *P*_st_ should occur at the point of turgor loss in the leaves (*T*_lp;_ refs. ^23,24^), for most species *P*_st_ approached less-negative values than the minimum *T*_lp_ for the species (Supplementary Figs. 6 and 7). Finally, *P*_st_ did not correspond to species-specific sensitivity to embolism formation (Supplementary Table 4 and Supplementary Figs. 6 and 7). We conclude that stomatal closure might only occur close to the *T*_lp_ under well-hydrated pre-dawn conditions (Supplementary Fig. 4), and the pre-dawn water status modulates commonly used *g*_s_ responses to midday *Ψ*_leaf_ and VPD^7^, particularly when pre-dawn *Ψ*_leaf_ values are more negative (Supplementary Fig. 8).

To test whether stomatal control is optimized to facilitate growth by sustaining turgor within the cambium^18^, we examined whether growth rates of the monitored trees at the Swiss Canopy Crane II (SCCII) site would halt at similar pre-dawn water status conditions as stomatal closure. Weekly manual readings of stem diameters, using band dendrometers, allowed us to relate tree-specific pre-dawn *Ψ*_leaf_ to daily growth rates driven by xylem and bark cell production and expansion^25^. Our findings showed that all species reduced stem growth rates to nearly zero after reaching −1.2 MPa pre-dawn *Ψ*_leaf_ (Fig. 2c), which corresponds to the water status at which midday stomatal closure occurs^20^. Additionally, the probability of growth across species rapidly decreased after −1.2 MPa, with only a 5% probability for any stem growth to occur after −1.5 MPa (Fig. 2c, *P* < 0.01). Indeed, growth cessation in herbaceous plants is often observed at less-negative water potentials^26^ than those reported in this study. One possible explanation is that acclimation through osmotic adjustments within cambial tissues may enable growth at more negative water potentials^27^, although this hypothesis warrants further investigation.

**Fig. 3:**
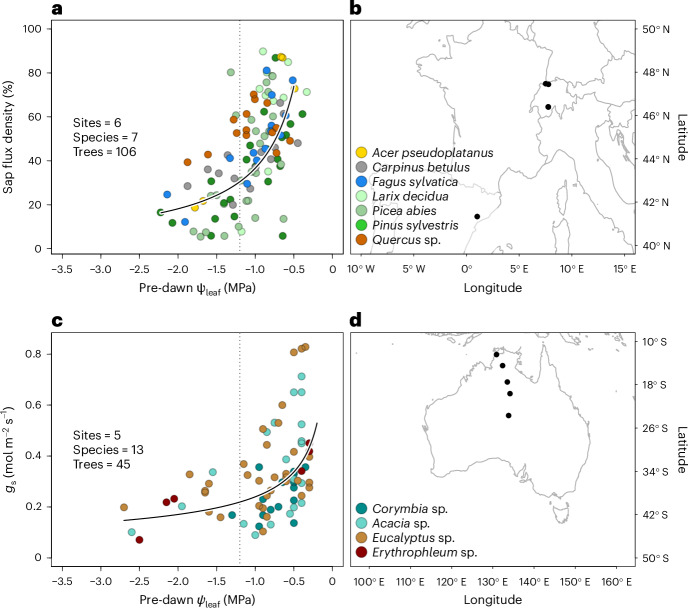
Sap flow and *g*_s_ response to pre-dawn *Ψ*_leaf_ across broad spatial scales. **a**, Pre-dawn *Ψ*_leaf_ measurements related to daily maximum sap flux density (expressed as a percentage of the maximum sap flux density of the tree). Species are distinguished by different colours. The line represents a significant log-transformed relationship, with species considered as a random effect to generate an across-species response curve. The grey dotted line is shown at −1.2 MPa for reference. **b**, Locations of the sites included in this analysis, as detailed in Supplementary Table 5. **c**,**d**, Relationship between pre-dawn *Ψ*_leaf_ and *g*_s_ for Australian tree species (**c**), distinguishable by colours as presented in the location map (**d**). Source data

To analyse whether pre-dawn *Ψ*_leaf_ plays a critical role in regulating whole-canopy *g*_s_, we compared the constraints imposed by pre-dawn and midday *Ψ*_leaf_ on whole-tree transpiration across multiple monitoring sites in Europe (Supplementary Table 5). We confirmed a strong relationship across species, showing that a more negative water status (that is, pre-dawn *Ψ*_leaf_ more negative than −1.2 MPa) significantly decreased sap flux density (*R*^2^ = 0.32, *P* < 0.0001; Fig. 3a,b). In contrast, midday *Ψ*_leaf_ did not predict a consistent or significant reduction in daily maximum sap flux density (*R*^2^ = 0.01, *P* = 0.218; Supplementary Fig. 9). Moreover, we again found a clear change in the hydroscape behaviour (or the relationship between pre-dawn and midday *Ψ*_leaf_), where a less steep decrease in midday *Ψ*_leaf_ with pre-dawn *Ψ*_leaf_ was found below −1.2 MPa (Supplementary Fig. 10), suggesting a clear shift in the whole-tree hydraulic response beyond this threshold. The importance of pre-dawn *Ψ*_leaf_ was also observed across four common tree genera growing in Australia along a steep precipitation gradient (278–1,705 mm of annual precipitation; Fig. 3c,d and Supplementary Table 6). Although full halt of conductance (*g*_s_ < 0.05 mol m^−2^ s^−1^) was not measured in these species, we nevertheless found again a shift in their stomatal behaviour and pre-dawn *Ψ*_leaf_ relationship at −1.2 MPa (ref. ^22^) (Supplementary Fig. 10). Despite expectations of *g*_s_ adjustment to drier conditions^28^, we found that across species *g*_s_ was substantially reduced with decreasing pre-dawn *Ψ*_leaf_ (*R*^2^ = 0.28, *P* < 0.0001), particularly when it approached −1.2 MPa.

Our data demonstrated that decreasing nocturnal water status of the entire tree plays a crucial role in inducing stomatal closure. Across natural forest sites, we found that pre-dawn *Ψ*_leaf_ is of primary importance for predicting *g*_s_ response, often forcing stomatal closure long before the midday water status reaches *T*_lp_ of leaves (Supplementary Fig. 7). Until now the −1.2 MPa pre-dawn *Ψ*_leaf_ threshold was solely reported in potted juvenile trees and shrubs^22,29^, without considering this in the context of midday *Ψ*_leaf_ or whether it would be a similar threshold for mature trees. The combined results we report here clearly indicate that pre-dawn water status is critically important for stomatal control across tree species, age classes and ecosystems. Here, we do, however, need to note that we still do not fully understand the exact interplay between midday VPD and pre-dawn *Ψ*_leaf_ on *g*_s_ (compare Supplementary Figs. 11 and 12).

Our results highlight that stomatal closure behaviour is not explained by the variance in vulnerability to xylem embolisms across species^8,17^. Instead, trees were observed to be conservative with their water usage, allowing stomata to remain open until the *T*_lp_ only when well hydrated in the morning (pre-dawn *Ψ*_leaf_ > −1.2 MPa; Supplementary Table 4). This behaviour is probably not easily detected in small trees, which are exposed to reduced water availability^8^ as a result of their smaller water-storage capacity, compared to tall trees, which makes the time between *T*_lp_ and the occurrence of xylem embolisms shorter. Larger trees probably benefit from greater storage capacity, which decreases the colinear behaviour between pre-dawn and midday water status^20^. The occurrence of stomatal closure when a mature tree stem cannot rehydrate over night can be physiologically explained, as negative pressures on the phloem hinder both sugar transport in the phloem and growth, and unconstrained water use until *T*_lp_ could cascade into ever-increasing water loss^13^. However, this does not diminish the importance of daytime conditions. Under well-hydrated pre-dawn conditions, mature trees appear to optimize stomatal closure according to species-specific osmotic adjustments in the leaf tissue^23^.

It is well-known that plant hormones such as abscisic acid (ABA) regulate stomatal closure^5,16^. However, the signalling pathways trees use to propagate hormonal response to a pre-dawn water status to control midday *g*_s_ response—as our data suggest—remain unclear. Leaf turgor has been identified as a key sensor within the signalling pathway^30^, yet this does not explain why stomata close long before leaf turgor is lost (Supplementary Fig. 7). Declining turgor associated with decreasing water potential of the leaves could still be important^31^. Alternative signalling could come from surrounding tissues, such as the mesophyll or phloem cells^17^, possibly through circadian processes that make these tissues more sensitive during night-time conditions. There is experimental evidence for high foliar ABA production in 8-year-old vines when the pre-dawn water status begins to decline^32^. However, the exact mechanisms by which tall trees perceive their water status in other tissues, such as the cambium of the stem, and at earlier times of day, remain unknown. The greater importance of pre-dawn water status reported here could explain why some studies on smaller plants emphasize the significance of root–soil connectivity for stomatal control^33^, as this is the primary process affecting rehydration in plants with lower water-storage capacity.

Water potential thresholds at which mature tree species close stomata are theorized to optimize carbon assimilation versus water availability in the living tissues, affecting other physiological functions such as growth. Current investigations of these thresholds for different tree species often focus on the instantaneous, daytime cost of turgor loss in the leaf tissue^8,23^. However, our findings suggest that pre-dawn water status in mature trees is probably more important. This underscores the necessity for trees to conserve water during the day to avoid compromising turgor-driven growth in the following night. Moreover, the inhibition of tissue formation at relatively high water potentials, closer to our observed pre-dawn threshold of −1.2 MPa than to the threshold of hydraulic failure, supports the importance of carbon sink activity in modulating stomatal conductance^26^. This dynamic diel controlling mechanism is currently not considered in most *g*_s_ modelling approaches, which typically simulate an instantaneous midday stomatal response (but see ref. ^13^). Our observed pre-dawn thresholds should thus be integrated into these models to avoid overestimating carbon assimilation at the landscape scale and beyond, particularly during drought events.

## Methods

### Study sites for detailed monitoring of gas exchange and sap flow

The majority of measurements were conducted in a mature mixed temperate forest at the SCCII site in Hӧlstein, Switzerland (47.439° N, 7.776° E, 500 m above sea level). Since 2018, the site has included a 50-m-tall canopy crane with a 62.5-m jib (Supplementary Fig. 2), allowing access to the canopies of over 300 trees via a manned gondola. Ecophysiological measurements were collected during 35 diurnal campaigns from 2020 until 2022, for a total of 95 trees (Supplementary Table 1). The tallest trees within the range of the crane from each of the nine species were selected as target trees. Diurnal campaigns were typically conducted from May until October, where each campaign included pre-dawn sampling before sunrise (04:00–06:00 Central European Time (CET)) and midday sampling (12:00–14:00 CET). In addition to the SCCII site, we included multiple European sites which have data on concurrent pre-dawn and midday *Ψ*_leaf_ combined with high-resolution sap flow measurements (Supplementary Table 6). Finally, we included a unique study along a North Australian climate gradient which collected *g*_s_ and both midday and pre-dawn *Ψ*_leaf_ for the genera *Eucalyptus*, *Corymbia* and *Acacia* (Supplementary Methods).

### Ecophysiological measurements and meteorological data

At the SCCII site, point measurements of *g*_s_ were made from branches in the upper canopy of the target trees. We used a LI-6800 Portable Photosynthesis System (LI-COR Biosciences), where for broadleaved trees, we selected healthy, sun-exposed leaves, whereas for conifers, we chose healthy sun-exposed second-year ramets to ensure fully developed leaves throughout the growing season. In Australia, point measurements of *g*_s_ were recorded using a Li-Cor 1600 Steady-State-Porometer (LI-COR).

Sap flow data collected from European forest sites (Supplementary Table 3) used thermal dissipation sap flow sensors, including either the SFS2‐M sensors (UP GmbH) or self-made sensors (for data processing see Supplementary Methods). All sensors were installed at a height of 1.5 m on the northeast side of the main tree bole, where dead bark was removed without damaging the phloem tissue. Raw measurements were obtained from either Hӧlstein or the corresponding authors of the published data (Supplementary Table 3), providing Δ*T* time series spanning periods where concurrent pre-dawn and midday *Ψ*_leaf_ measurements were performed.

At all sites, measurements of *Ψ*_leaf_ were performed by using a Scholander-type pressure chamber (PMS Instrument Company). All measurements were conducted immediately after sampling of sun-exposed apical branches with multiple healthy leaves attached to each one. In June and August of 2023, we sampled additional branches to generate pressure–volume curves with which we established tree-specific leaf turgor loss points (*T*_lp_).

At the SCCII site, band dendrometers (D1 tree girth band, Meter GmbH) were mounted at breast height (1.3 m above the ground) on the stems of all target trees (Supplementary Table 1). Before mounting, the stem surface was cleaned of other vegetation and uneven parts of the outer bark. Approximately weekly readings were performed manually throughout the monitoring years to record the diameter at breast height (DBH in cm). Moreover, to confirm the accuracy of the band dendrometers (ZN11-T-WP type, Natkon), for some of the target trees point dendrometers were installed which automatically recorded radial changes in a higher precision and a temporal resolution of 10 min.

On-site air temperature (*T*_a_ in °C), relative humidity (%), solar irradiance (W m^−2^) and precipitation (mm) were monitored using a weather station placed in a forest gap at the SCCII site (Davis Vantage Pro 2, Scientific Sales). Additional measurements of solar irradiance (W m^−2^) and precipitation (mm) were collected from the top of the crane using the same climate station, where precipitation patterns were averaged over all sensors.

### Data treatment and statistical analyses

The relationship between *Ψ*_leaf_ and *g*_s_ was tested using linear mixed-effect modelling. This process involved model selection, model assumption testing, and finally, post hoc tests and model application. Absolute *g*_s_ values vary between species and can introduce issues when performing analyses that include multiple species. For this reason, we normalized our *g*_s_ data to the maximum species-specific *g*_s_ values. We performed a log transformation on both the *g*_s_ measurements and VPD (measured by the LI-6800) to better describe the nonlinear behaviour and avoid violating normality assumptions for the residuals. Tree number was included as a random effect, while species was added as a fixed effect.

To determine the point of stomatal closure (*P*_st_) due to *Ψ*_leaf_, we used the linear mixed-effect model to predict the linear part of the data after stomatal closure (at more negative *Ψ*_leaf_ values), where VPD conditions are set to high values (VPD = 2.4 kPa; Supplementary Fig. 5). When the data points approach the 95% confidence interval of this projection under high VPD conditions, we define this as the *P*_st_ as the behaviour becomes statistically indistinguishable from the approached linear decrease (schematically shown in Fig. 1c). To generalize the behaviour of all data, we used a generalized additive mixed-effect model (GAMM) function. The intercept between the 95% confidence interval and the GAMM model was used to represent the point when stomatal behaviour reaches an inflection point, transitioning from active closure to approaching *g*_s_ reduction due to loss of conductivity to soil water reservoirs. Moreover, we tested the robustness of the *P*_st_ by using the fitted GAMM to quantify the *Ψ*_leaf_ point at which *g*_s_ was <0.05 mol m^−2^ s^−1^, a common stomatal closure threshold used in literature^21^ (Supplementary Fig. 4). We modelled the stomatal response to pre-dawn *Ψ*_leaf_ for both sap flow-derived stomatal conductance and *g*_s_ measurements from Australia using a linear mixed-effects model with log-transformed dependent and independent variables, treating species and site as random effects.

Growth rates were calculated by determining the difference in DBH between each monitoring session and dividing this by the number of days between measurements. We applied the zero-growth concept to the DBH data before calculating the radius growth rates, to ensure that no negative growth will be included in the analyses (due to drought induced shrinkage). For the analyses we only considered June, July and August, to avoid the inclusion of growth halt due to winter dormancy of the cambium. A generalized linear mixed-effect model with a binomial distribution was applied to assess the probability of growth occurrence across species, using species and tree nested in species as random effects. See Supplementary Fig. 13 for the results from the daily point dendrometer measurements. More detailed information on the statistical methods is provided in the Supplementary Methods.

### Reporting summary

Further information on research design is available in the Nature Portfolio Reporting Summary linked to this article.

## Supplementary information

Supplementary Information Supplementary methods, Figs. 1–14 and Tables 1–6.

Reporting Summary

## Source data

Source Data Fig. 1 Statistical source data.

Source Data Fig. 2 Statistical source data.

Source Data Fig. 3 Statistical source data.

## Data Availability

Data are available in the main text and Supplementary Information. The gas exchange, sap flow, leaf water potential and growth data used in this study are available via Zenodo at 10.5281/zenodo.14852038 (ref. ^34^). Source data are provided with this paper.
