# Supplementary material for: Uniform regulation of stomatal closure across temperate tree species to sustain nocturnal turgor and growth
Source: Nat Plants. 2025 Apr 3;11(4):725–30. doi: 10.1038/s41477-025-01957-3 (PMC12014480; doi:10.1038/s41477-025-01957-3)
Supplement: Supplementary file 1 — Supplementary methods, Figs. 1–14 and Tables 1–6. [file 41477_2025_1957_MOESM1_ESM.pdf]

nature plants

Supplementary information https://doi.org/10.1038/s41477-025-01957-3

Uniform regulation of stomatal closure across temperate tree species to sustain nocturnal turgor and growth

In the format provided by the authors and unedited

Uniform regulation of stomatal closure across temperate tree species to sustain nocturnal turgor and growth

Richard L. Peters, Matthias Arend, Cedric Zahnd, Günter Hoch, Stefan K. Arndt, Lucas A. Cernusak, Rafael Poyatos, Tobias Zhorzel & Ansgar Kahmen

Supplementary Methods

Overview and research concept

In-situ measurements of the reduction in g_s due to drought across a diverse range of tree species are critical for supporting our expanding modelling efforts[1]. We used a 50 m tall canopy crane at the Hölstein research site in Switzerland, with a 62.5 m long jib, to monitor stomatal conductance (g_s) and leaf water potentials (Ψ_{leaf}) in the crowns of over 95 mature trees from 9 common European species (>1000 measurements; Supplementary Table 1, 2). During 35 sampling dates from 2020 to 2022, we measured concurrent midday (12:00 – 14:00 CET) g_s and Ψ_{leaf} from the same individual trees (Supplementary Fig. 3), as well as pre-dawn (04:00 – 06:00 CET) Ψ_{leaf} to establish tree hydration status (Fig. 1). The relatively dry 2022 growing season contrasted with the wet 2021 conditions (Supplementary Fig. 1), providing a unique, well-replicated range of tree-internal water status (Fig. 1b). We analyzed the relationships between g_s and Ψ_{leaf} alongside in-situ vapor pressure deficit (VPD), accounting for the effects of species and repeated measurements per tree. Additionally, we compiled a larger dataset on sap flow measurements and g_s, concurrently measured with pre-dawn and midday Ψ_{leaf}, to validate our findings at other sites (Supplementary Table 6). Using this dataset, we aimed to: (i) assess the sensitivity of mature tree g_s regulation to pre-dawn and midday Ψ_{leaf}, (ii) disentangle the active and species-specific thresholds driving stomatal closure, and (iii) investigate the link between Ψ_{leaf} and growth rates measured with band dendrometers.

Canopy crane site description and sampling design

Measurements were conducted at the Swiss Canopy Crane II (SCCII) site in Hölstein, Switzerland (47.439 °N, 7.776 °E, 500 m a.s.l.). The site consists of a mature mixed temperate forest growing on a clay-rich soil over limestone. The 1.68 ha research area comprises of 476 living trees (diameter at breast height, d_{stem} > 10 cm) from 14 different species (Supplementary Fig. 2). The leaf area index is approximately 2.2 m m^{-2}, with a mean d_{stem} of 31 cm (range 10 – 80 cm) and a mean tree height (h_{tree}) of 24 m (range 4 – 36 m; see [2] for a more detailed site description). The mean annual temperature and precipitation at the site are 9.6 °C and 972 mm, respectively (mean data 1991-2020, MeteoSwiss climate station

Rünenberg). Since 2018, the site has included a 50 m tall canopy crane with a 62.5 m jib installed in the middle of the site (Supplementary Fig. 2), allowing access to the canopies of over 300 trees via a manned gondola. On-site air temperature (T_a in °C), relative humidity (%), solar irradiance (W m^{-2}), and precipitation (mm) were monitored using a weather station placed in a forest gap (Davis Vantage Pro 2, Scientific Sales Inc., Lawrenceville, NJ, USA). Additional measurements of solar irradiance (W m^{-2}), and precipitation (mm) were collected from top of the crane using the same climate station, with precipitation patterns were averaged over all sensors. Relative humidity and T_a were used to calculate VPD (kPa) using the "*plantecophys*" R package[3]. All the environmental monitoring data was collected at 10-minute intervals.

Eco-physiological measurements were collected during 35 diurnal campaigns from 2020 until 2022, utilizing the crane to access the tree canopies. In total, 95 trees were measured (Supplementary Table 1), including broadleaved species such as *Fagus sylvatica* L., *Acer pseudoplatanus* L., *Fraxinus excelsior* L., *Carpinus betulus* L., and *Sorbus torminalis* Crantz, and conifers *Picea abies* Karst., *Abies alba* Mill. and *Pinus sylvestris* L. Additionally, *Quercus* trees, which were hybrids of *Quercus petraea* Liebl. and *Quercus robur* L., to various degrees, were treated as a single species for this study. The tallest trees within the crane's range from each of the nine species were selected as target trees. Target trees in some cases changed due to crown damage from drought events, the need to avoid excessive pruning, or other projects at the research site (Supplementary Table 1). For each target tree we recorded multiple characteristics, including diameter at breast height (d_{stem}), tree height (h_{tree}; using a Vertex IV, Haglöf, Sweden), exact location (longitude and latitude), health status (i.e., alive vs. dead), and leaf phenology status (according to [4]). Diurnal campaigns were typically conducted from May until October, when leaves were fully developed (Fig. 1). Each campaign included pre-dawn sampling before sunrise (04:00 – 06:00 CET) and midday sampling (12:00 – 14:00 CET). During pre-dawn sampling, only leaf water potential (Ψ_{leaf}) measurements were performed, while at midday, both Ψ_{leaf} and stomatal conductance (g_s) were recorded.

Data collection from other sources

Multiple studies have presented data of concurrent pre-dawn and midday Ψ_{leaf} combined with high-resolution sap flow measurements in Europe (Supplementary Table 6). In addition

to the sap flow data collected from the target trees at the SCCII site in Hölstein in 2022, measurements were also obtained from Hofstetten, Switzerland[5]. The Hofstetten site is situated in a diverse mixed forest at an elevation of 550 m a.s.l., with a rocky rendzina-type soil based on calcareous bedrock. The forest comprises both evergreen and deciduous species, predominantly *Fagus sylvatica* and *Quercus petraea*. The trees at this site are approximately 130 years old and between 35 and 40 m in height. A canopy crane was employed to access the tree crown for Ψ_{leaf} measurements. The mean annual temperature at Hofstetten is 10.5°C, and the mean annual precipitation 990 mm. From this study site, we utilized data collected in 2014 and 2015, which are detailed further in [5].

We further utilized sap flow data from three sites situated in the Swiss Lötschental valley[6]. Two of these sites are situated at 1300 m a.s.l., featuring contrasting dry and wet soil conditions due to the presence of the Lonza river. The third site is located near the treeline at 2200 m a.s.l., characterized by colder growing season conditions. The valley features steep slopes (>60%) and soils formed from calcareous-free substrate, including moraines and crystalline bedrock (gneiss and granite) from the Aar massif. The soil types vary from Leptosol, Cambisol to Podzol. These slopes are covered with a mixed forest of naturally occurring evergreen *Picea abies* and deciduous *Larix decidua*. The trees are approximately 170 years old and around 22 m tall. Samples for Ψ_{leaf} measurements were collected with a 3 m long pole pruner. The long-term mean annual temperature in the valley is 5.7 °C and the mean annual precipitation 800 mm. Data from these three sites, collected in 2014 and 2015, are detailed further in [7].

The southern most European site used for this study is located in the Tillar valley within the Poblet nature reserve (Prades Mountains, northeast Spain[8]). The Tillar site is located on a slope (>30%) within a valley where coppiced woodland occupy the lower elevations, while higher elevations (1015 m a.s.l. and above) are dominated by *Pinus sylvestris* stands. The site is characterized by fractured schist resulting in fairly rocky and shallow xerochrept soils with a loamy texture. The *Pinus sylvestris* population is at least 150 years old, with trees reaching a height of 14 m. Canopies were accessed using a pole pruner for Ψ_{leaf} measurements. The mean annual temperature at the site is approximately 11.3 °C, with a mean annual precipitation of 664 mm. Sampling and continuous sap flow measurements were conducted in 2010 and 2011, as detailed in [8].

A unique dataset along a North Australian climate gradient was made available, featuring concurrent measurements of midday (14:00 – 15:00) g_s and both midday and pre-dawn Ψ_{leaf}[9]. The dataset includes five sites spanning a large precipitation gradient, from 1705 mm of annual precipitation in the north (Darwin in Supplementary Table 6) to 278 mm in the south (Alice springs in Supplementary Table 6). Sites in the south were at higher elevations, with an elevational range from 33 m to 598 m a.s.l. from north to south. The transect traverses from a mesic (>1200 mm annual rainfall) to a semi-arid savanna and into an arid zone (see [9] for a more detailed description). The vegetation primarily consists of species belonging to the two dominant Australian genera, *Acacia* and *Eucalyptus*. Along the precipitation gradient, a mix of species from the genus *Eucalyptus*, *Corymbia*, and *Acacia* was measured during two periods of the growing season. Crowns were accessed using a pole pruner.

Ecophysiological measurements

During a diurnal campaign, point-measurements of g_s were made from branches in the upper canopy of target trees (Supplementary Table 1). We used a LI-6800 Portable Photosynthesis System (LI-COR Biosciences GmbH, Bad Homburg, Germany), which was taken into the crane gondola and quipped with either a broadleaf small light source chamber or a conifer chamber with a large light source. For broadleaved trees, we selected healthy, sun-exposed leaves, whereas for conifers, we chose healthy sun-exposed second-year ramets to ensure fully developed leaves throughout the growing season. When performing a measurement, the temperature and relative humidity were set to ambient conditions, and light was provided at 1000 μmol m^{-2} s^{-1} with a 30% red and 70% blue ratio to mimic natural light conditions. All other settings of the LI-6800 device were performed according to the operational manual. For the conifers, the leaf area parameter in the processing files provided by LI-COR was corrected for the actual needle area in the chamber. This correction was achieved by measuring the one-sided projected needle area, determined by carefully spreading the needles from the measured ramet onto a flat-bed scanner (Epson Expression 12000XL; Epson, Nagano, Japan). The needle area was then extracted using a dedicated digital image analysis tool (github.com/dabasler/LeafAreaExtraction). In Australia, point-measurements of g_s were recorded using a Licor 1600 Steady-State-Porometer (LiCor Inc., Lincoln, Nebraska, USA). These measurements were performed on a sun-exposed leaf for

each tree, with each of the five sites being visited at two specific times in the growing season: March to April 2010 and September 2010.

Measurements of Ψ_{leaf} were performed by using a Scholander-type pressure chamber (PMS Instrument Company, Albany, Oregon, USA). For each tree, we selected 2 to 3 small branches, 5-10 cm long, with multiple healthy sun-exposed leaves or needles attached to it. Direct measurements were specifically performed on the leaf and petiole of *Fraxinus excelsior* because the xylem in this species is large enough to clearly identify the point at which water exhausts from the xylem tissue. After sampling the branches were shortly stored in a bag (less than approximately 20 minutes per sample) while bringing the samples down from the canopy. All measurements were conducted immediately after the previously described sampling using the canopy crane.

Pressure-volume (PV)-curves were constructed in June and August during the growing season 2023 to determine leaf turgor loss points (T_{lp}) of the target trees. Branches were cut in the morning, stored in cooling boxes, and transported to the laboratory, where they were recut under water and rehydrated to full turgor after which a bench drying method was applied.

Band dendrometers (D1 tree girth band, Meter GmbH München, Germany) were mounted at breast height (1.3 m above the ground) on the stems of all target trees (Supplementary Table 1). Before mounting, the stem surface was cleaned of other vegetation and uneven parts of the outer bark. Approximately weekly readings were performed manually throughout the monitoring years to record the diameter at breast height (DBH in cm). The timeseries obtained from weekly band dendrometer readings were used as unprocessed data, except in cases where obvious outliers due to mis-readings were identified. Point dendrometers (ZN11-T-WP type, Natkon, Oetwil am See, Switzerland) were installed on the target trees at breast height (1.3 m above the ground) on the north-facing side of the tree stem. Before installation, the stem surface was prepared by carefully removing dead bark while avoiding damage to the underlying living bark. This ensured a smooth surface close to the phloem tissue and minimized hygroscopic swelling due to rainwater. The dendrometers consisted of a carbon-fiber frame, which was attached to the stem using three stainless steel threaded rods anchored approximately 5 cm deep into the wood tissue. The metal sensing rod was gently placed on the prepared bark tissue to measure stem radius changes. Data were collected using a DecentLab data logger (DecentLab GmbH, Dübendorf, Switzerland) and

stored at a 10-minute resolution. The logging resolution was <1 μm, and the temperature sensitivity of the dendrometers was <0.3 μm °C-1.

Sap flow data collected from European forest sites (Supplementary Table 3) used thermal dissipation sap flow sensors, including either the SFS2-M sensors (UP GmbH, Ibbenbüren, Germany) or self-made sensors following the[10] protocol. All sensors were installed at a height of 1.5 m on the north-east side of the main tree bole, where dead bark was removed without damaging the phloem tissue. Using a drill, two 20 mm long probes were radially inserted into the xylem (beneath the cambium) with a vertical distance of 10 cm between them and shielded from direct sunlight. The temperature difference between the heated and unheated probe (ΔT in °C) was recorded every 10-30 minutes using a sensor node at the research site. Raw measurements were obtained from either Hölstein or the corresponding authors of the published data (Supplementary Table 3), providing ΔT time-series spanning periods where concurrent pre-dawn and midday Ψ_{leaf} measurements were performed. The ΔT measurements from sap flow sensors were converted to sap flux density (F_d; kg m^{-2} s^{-1}) using the TREX R package[11]. This conversion involved: i) applying the double-regression method to establish zero-flow conditions (using a 5-day period), ii) making sapwood corrections (using sapwood thickness measurements or estimations), and iii) applying species- (*F. sylvatica*, *P. abies* and *L. decidua*) or wood-specific (*Pinus sylvestris* = Coniferous, *Quercus petraea*/*sp.* = Ring-porous and *Carpinus betulus* = Diffuse-porous; *Acer pseudoplatanus* = Diffuse-porous) calibrations, as calibration studies were not present for all species. After inspecting for outliers, all data were averaged to hourly timesteps and normalized to the 99th percentile of maximum F_d to enhance comparison between tree species (according to [5]).

All monitoring data were inspected for outliers using the *datacleanr* package[12] in the R software environment (version 4.2.2, R Core Team 2022). For the g_s data, we removed negative measurements, as these could be caused by measurement errors or other physiological processes not relevant to our study on stomatal closure. Additionally, log-transformation required for further data analysis does not accommodate negative values. We also excluded unrealistically high conductance values for specific species, which occasionally occurred during periods with relatively high relative humidity and low temperature settings of the LI-6800 due to ambient conditions. Moreover, unrealistically high conductance values compared to all other measurement values was removed from the LI-1600 data series.

Environmental monitoring data and sap flow data was inspected for outliers using the *datacleanr* package. Erroneous data points include drastic single outlier values, jumps, and random noise generate by sensor failure.

Data treatment and statistical analyses

The data processing and statistical analyses were performed in the R programming environment. Linear mixed-affect models were formulated using the package "nlme"[13] and "lme4"[14] and post-hoc tests were done using the package "emmeans"[15].

The relationship between Ψ_{leaf} and g_s we tested using linear mixed-effect modelling. This process involved model selection, model assumption testing, and finally post-hoc tests and model application. Absolute g_s values vary between species and can introduce issues when performing analyses that include multiple species. Therefore, it is common practise to test both raw g_s responses and normalized values[16]. We followed this approach by normalizing our data; each g_s value was divided by the maximum g_s value recorded for the species. This maximum value was excluded from the analyses to avoid data inflation towards the value 1. We did not perform a tree-specific normalization as we could not assure that we measured the absolute maximum conductance for each tree, due to sampling range differences in time.

For the model selection, we used the linear model described in *Eq. 1* as a starting point. We performed a log transformation on both the g_s measurements and VPD (measured by the LI-6800) to better describe the non-linear behaviour and avoid violating normality assumptions for the residuals. Tree ID was included as a random effect, while species was added as a fixed effect.

$$\log(g_s)_{ij} = \beta_0 + \beta_1 \cdot \varphi_{\text{leaf}_{ij}} \cdot \text{species}_j + \beta_2 \cdot \log(\text{VPD})_{ij} \cdot \text{species}_j + u_i + \epsilon_{ij} \qquad \textit{Eq. 1}$$

Within *Eq. S1* $\log(g_s)_{ij}$ represents the observed logarithm of stomatal conductance for data point *i* within tree *j*. φ_{leaf} is either pre-dawn or midday leaf water potential for data point *i* in tree *j*. *VPD* is the log-transformed vapor pressure deficit for data point *i* within tree

j. The variable species_j is an indicator for the species of tree associated with tree j, introducing a species-specific effect. β_0 is the intercept, capturing the population average effect of log(g_s). β_1 and β_2 are slope coefficients for the independent variables φ_{leaf} and log(VPD), respectively, while considering the interaction with species. u_i is the tree-specific random effect, accounting for the variation within a tree (random intercept). ϵ_{ij} is the error term, representing the residual variation not explained by the model and assumed to have a Gaussian distribution. Model selection was performed by including or excluding interactions or variables by using the Akaike information criterion and logical reasoning.

The selected models were tested for their assumptions by systematically inspecting for the following: 1) Outliers, 2) Homogeneity, 3) Normality, 4) Zero trouble, 5) Collinearity, 6) Relationship, 7) Interactions, and 8) Independence in accordance with[17]. We did not have sufficient data to incorporate the interaction between pre-dawn and midday Ψ_{leaf}, as their dynamics are highly collinear. All models presented in this manuscript did not show clear signs of violating the above-mentioned assumptions.

Besides performing model fitting and testing on the entire dataset, we also isolated data where trees were well hydrated (pre-dawn $\Psi_{\text{leaf}} > -1$ MPa). This isolation was performed to focus on the response of midday g_s to midday Ψ_{leaf} or VPD. In these analyses, either midday Ψ_{leaf} or VPD was selected as a single independent variable, along with species, while considering the tree as a random effect. All described models were used the predict the dependent *vs*. independent relationship by using the "*emmeans*" R package.

To determine the point of stomatal closure (P_{st}) due to Ψ_{leaf}, we initially attempted to perform a piecewise linear regression (or "broken stick regression") as is commonly done to identify the approximate point of stomatal closure[18]. However, due to the large number of data and the continuous and exponential nature of the relationship between Ψ_{leaf} and g_s, the piecewise regression did not produce clearly isolated segments. Therefore, we resorted to utilizing the linear mixed-effect model as explained in *Eq. 1* (see also Supplementary Table 5). We used this model to predict the linear part of the data after stomatal closure (at more negative Ψ_{leaf} values), where VPD conditions are fixed to high values using *emmeans* (VPD = 2.4 kPa; see Supplementary Fig. 5). The assumption here is that this linear decrease is driven by minimal leaf conductance and root conductivity of the plant[19,20]. When the data points approach the 95% confidence interval of this projection under high VPD conditions, we define this as the P_{st} as the behaviour becomes statistically undistinguishable from the

approached linear decrease. To generalize the behaviour of all data, we used a generalized additive mixed-effect model (GAMM) function from the "*mgcv*" R package[21]. The intercept between the 95% confidence interval and the GAMM model was used to represent the point when stomatal behaviour reaches an inflection point, transitioning from active closure to approaching *g*s reduction due to loss of conductivity to soil water reservoirs. Moreover, we tested the robustness of the P_{st} by using the fitted GAMM to quantify the Ψ_{leaf} point at which g_s was at 0.04 mol m^{-2} s^{-1}, as <0.05 mol m^{-2} s^{-1} is a common stomatal closure threshold used in literature[22]. To further confirm the validity of the P_{st}, we analysed the hydroscapes[20] where we tested the relationship between pre-dawn and midday Ψ_{leaf} and how it changes before and after P_{st}. The general relationship between pre-dawn and midday Ψ_{leaf} was described by using a LOESS smoother on raw data.

To ensure that our found stomatal closure points to pre-dawn ψ_{leaf} are not confounded by the collinearity between VPD and pre-dawn ψ_{leaf} we performed more detailed analyses. We explored the collinearity issues between pre-dawn ψ_{leaf} and VPD by plotting the response of pre-dawn ψ_{leaf} to VPD and confirm that the response is not linear (Supplementary Fig. 11a). Although a high Spearman's Rank Correlation of 0.6 was found, the non-linearity of the relationship causes the Variance Inflation Factor (VIF) test of our model (explaining g_s response with both VPD and pre-dawn ψ_{leaf}; *Eq.* 1) to be below 4, indicating little problematic collinearity between the variables. Moreover, most high VPD values, at low pre-dawn ψ_{leaf}, did not reach *g*s values close to stomatal closure (Supplementary Fig. 11b). To visualize the importance of pre-dawn ψ_{leaf}, we build a model with solely the impact of log transformed VPD on log transformed g_s (removing ψ_{leaf} in *Eq.* 1). We used this model for each species to remove the VPD effect and focus on the residuals. These residuals were tested against pre-dawn ψ_{leaf} to see the relationship with these residuals of g_s (Supplementary Fig. 12). When performing fitting a linear mixed-effect model with the trees nested in species the overarching fit was significant ($P = 0.005$), indicating the importance of pre-dawn ψ_{leaf} independent from the midday VPD response.

Growth rates were calculated by determining the difference in DBH between each monitoring session and dividing this by the number of days between measurements. Although rare, we did record negative increments during days with severe droughts. This can occur due to the negative tension on the water column in the xylem causing the bark tissue to shrink. To prevent these measurements from affecting the analyses, we applied the zero-growth concept

to the DBH data before calculating the rates[23]. We matched the daily growth rates (expression in µm radius per day) to the Ψ_{leaf} data by identifying the closest monitoring data to the sampling date. We assumed growth rates to be constant when the DBH monitoring was performed within 10 days, excluding points with a longer monitoring interval. For the analyses we only considered June, July, and August, to avoid the inclusion of growth halt due to winter dormancy of the cambium. A generalized-linear mixed-effect model with a binomial distribution was applied to assess the probability of growth occurrence across species, using species and tree nested in species as random effects. For the point dendrometer measurements we used the matching daily growth rates and performed similar modelling to confirm the patterns found with the longer time-series of band dendrometers (Supplementary Fig. 13). Species-specific growth to pre-dawn ψ_{leaf} fits are only presented for the band dendrometers due to low replication of the point dendrometer measurements. Moreover, due to the low number of zero-growth values and small deviations within the band dendrometer readings value below 10 µm d^{-1} were considered zero-growth within the growth probability analysis (see Supplementary Fig. 14).

For the determination of T_{lp} of the target trees, fully turgid samples were weighed to calculate the relative water content (RWC= (fresh weight – dry weight)/(turgid weight – dry weight)). PV-Curves were constructed based on consecutive measurements of the RWC and $1/\Psi_{leaf}$[24]. To ensure a good characterization of the initial drop in Ψ_{leaf}, samples were placed into humid plastic bags. A non-linear model was fit using R based on the approach developed by Schulte & Hinckley 1985[25]. The TLP was defined as the point where the curve switches from being defined only by the osmotic potential (linear part) to being defined by the osmotic potential and turgor potential (non-linear part).

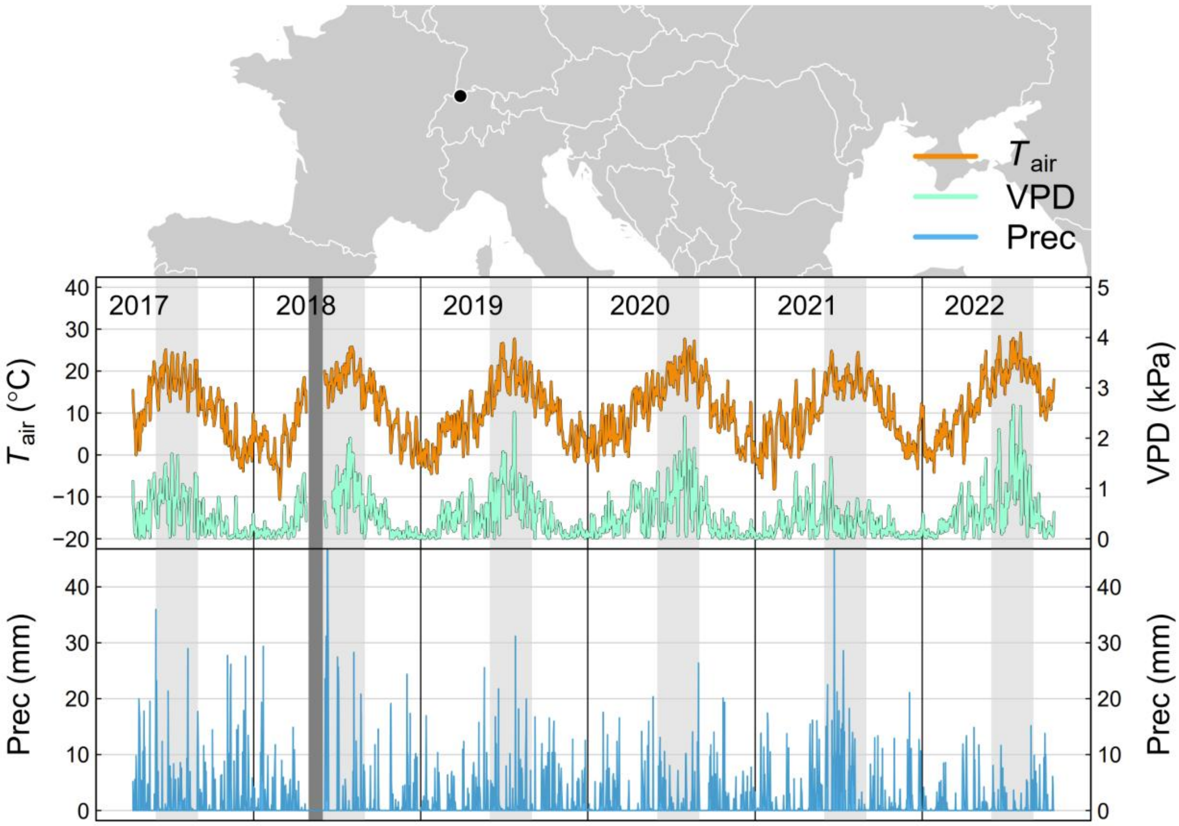

Supplementary Figure 1. Climatic conditions during the 2017 to 2022 monitoring period at the Swiss Canopy Crane II (SCCII) site in Hölstein, Switzerland. Daily mean atmospheric temperature (T_{air}) and vapor pressure deficit (VPD) were measured with a climate station at the SCCII site. The dark grey area indicates a data gap in 2018. Daily summed precipitation (Prec) dynamics measured at the site are also shown. The grey areas in the plot are the months June, July and August. The black dot in the map indicates the study site.

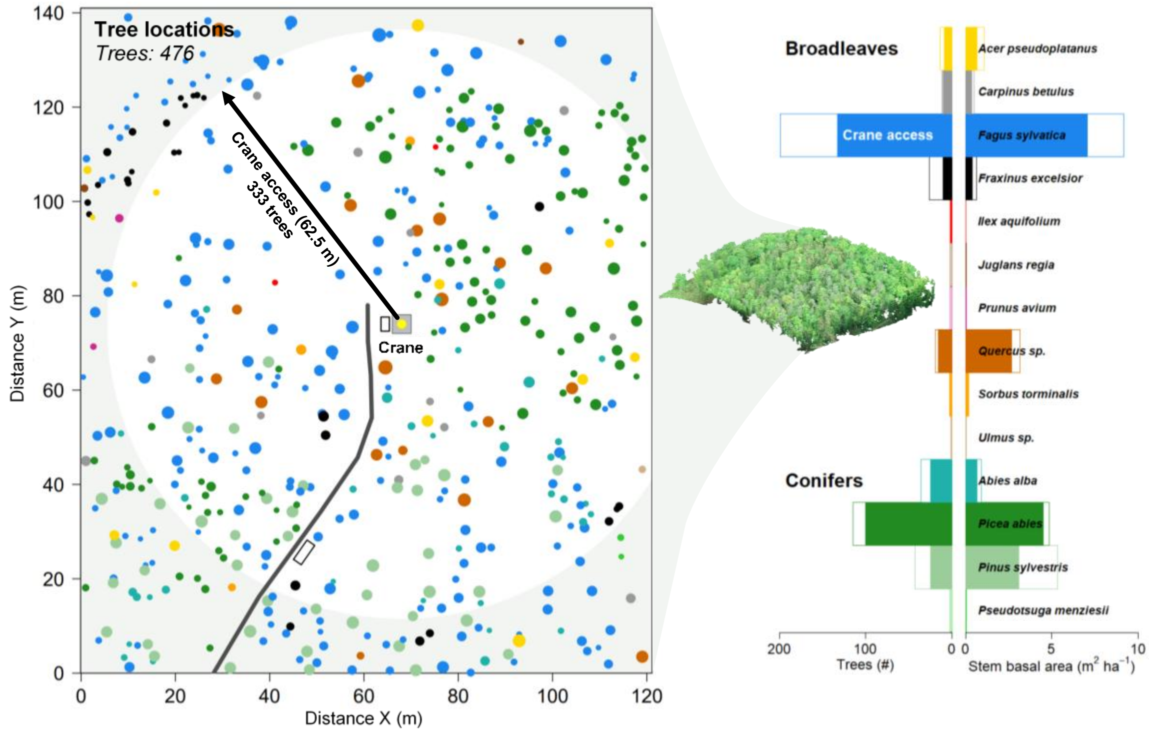

Supplementary Figure 2. Site map of the Swiss Canopy Crane II (SCCII) site in Hölstein, Switzerland. The map shows the locations of the trees, with dot size indicating the relative diameter at breast height (d_{stem}). Each color presents a species, as indicated in the accompanying bar graph. The bar graph provides information on the number of trees (d_{stem} > 10 cm) and the stem basal area per species. The colored-in bars highlight the number of trees that are accessible by the canopy crane.

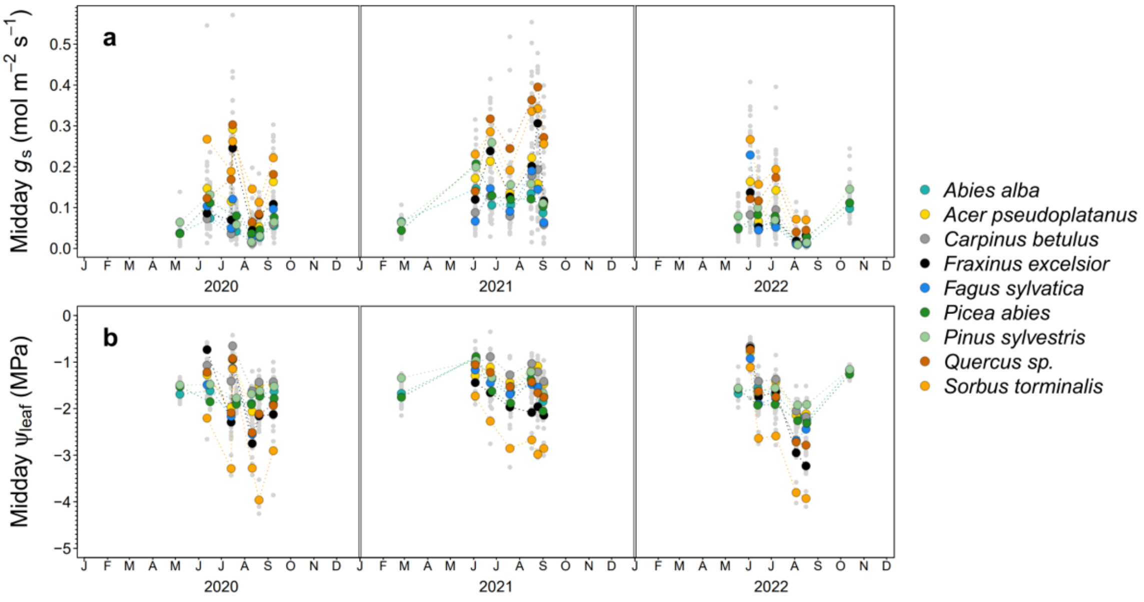

Supplementary Figure 3. Time series of stomatal conductance (g_s) and midday leaf water potentials (Ψ_{leaf}). (a) Mean species-specific midday g_s measured on leaves is presented with the large colored circles. White dots indicate measurements dates where Ψ_{leaf} was more negative than -1.2 MPa. The colors represent different species, as indicated in the legend. (b) Midday (12:00 – 15:00 CET) Ψ_{leaf}, with large colored circles representing the species mean.

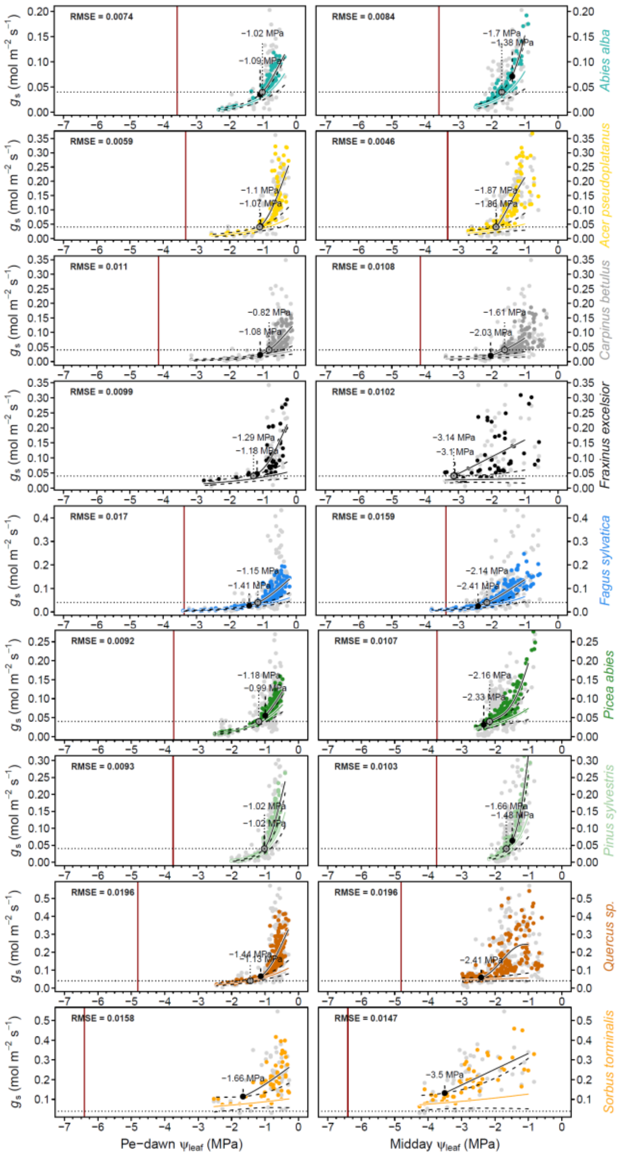

Supplementary Figure 4. Species-specific midday g_s response to pre-dawn and midday Ψ_{leaf}. The model described in Supplementary Table 5 disentangles the g_s-Ψ_{leaf} response when *D* is high (*D* = 2.4 kPa) to isolate the impact of water potential impact (colored line, with dashed line showing the 95% confidence interval). A GAMM describes the behavior of the data when only considering Ψ_{leaf} as an independent variable (solid black line). The point where the solid black line crosses the upper confidence interval of the full model (at *D* = 2.4 kPa) is considered the point of stomatal closure (P_{st} in Fig. 2). As a robustness check, we also marked the point when the GAMM model crossed a fixed low g_s value (<0.05 mol m^{-2} s^{-1}), indicated with the grey-filled circle. Mind that *Quercus* and *Sorbus* did not always show such low values. The dark red line indicates the species-specific point of 50% loss of hydraulic conductivity (P_{50}).

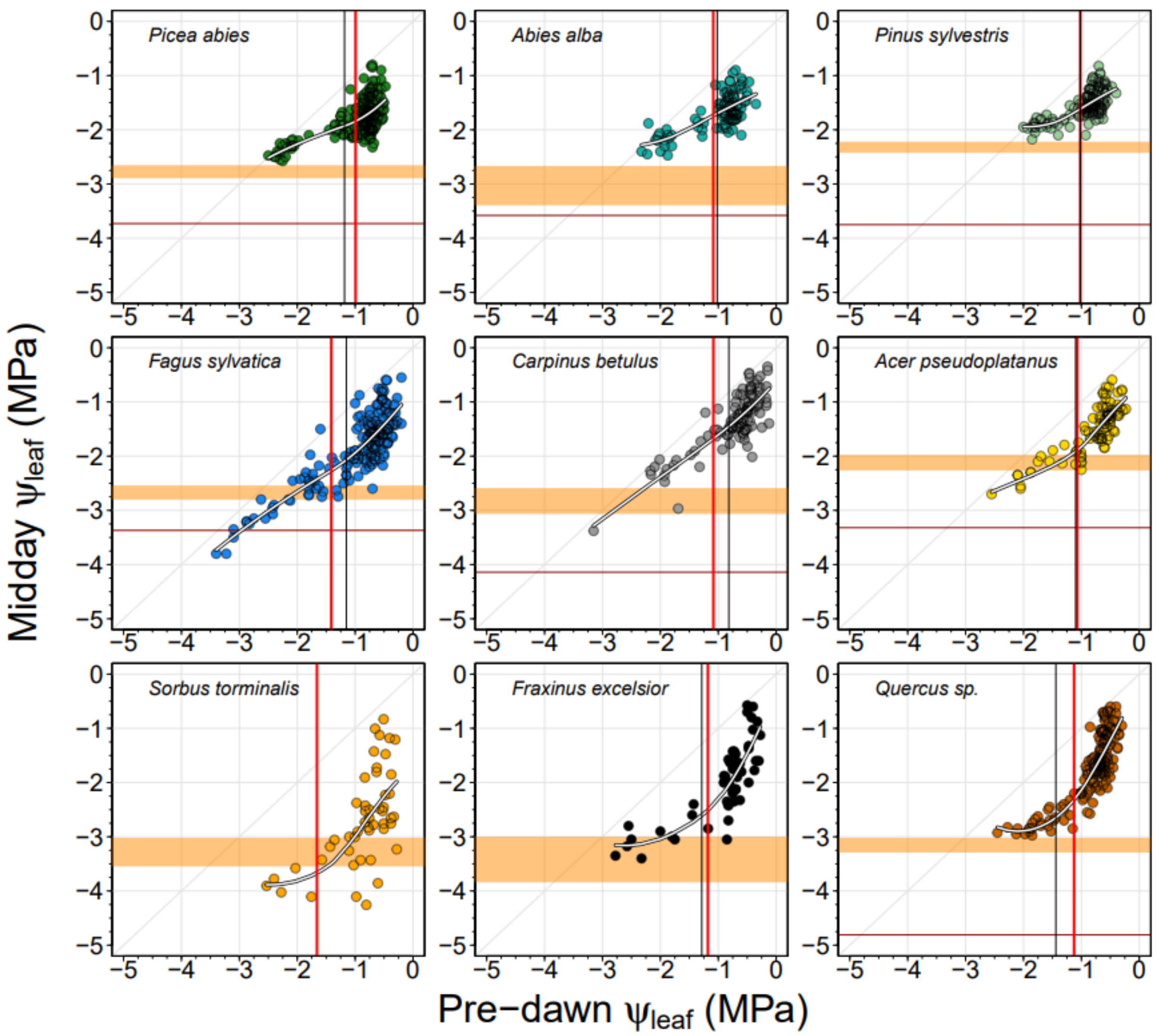

Supplementary Figure 5. Relationship between pre-dawn and midday Ψ_{leaf} and its association with drought-induced stomatal closure. For each species, raw data points of midday and pre-dawn Ψ_{leaf} are presented, with their relationship shown using a LOESS smoother (white bold lines). The red lines indicate the P_{st} for pre-dawn Ψ_{leaf} conditions per species as presented in Fig. 1c, while the black lines indicate the pre-dawn boundary when g_s is below 0.05 mol m^{-2} s^{-1}. The orange shaded area shows the confidence interval of the Ψ_{leaf} values at which leaf turgor is lost, while the dark red line indicates the point at which 50% of hydraulic conductivity is lost (P_{50}; see Supplementary Table 4).

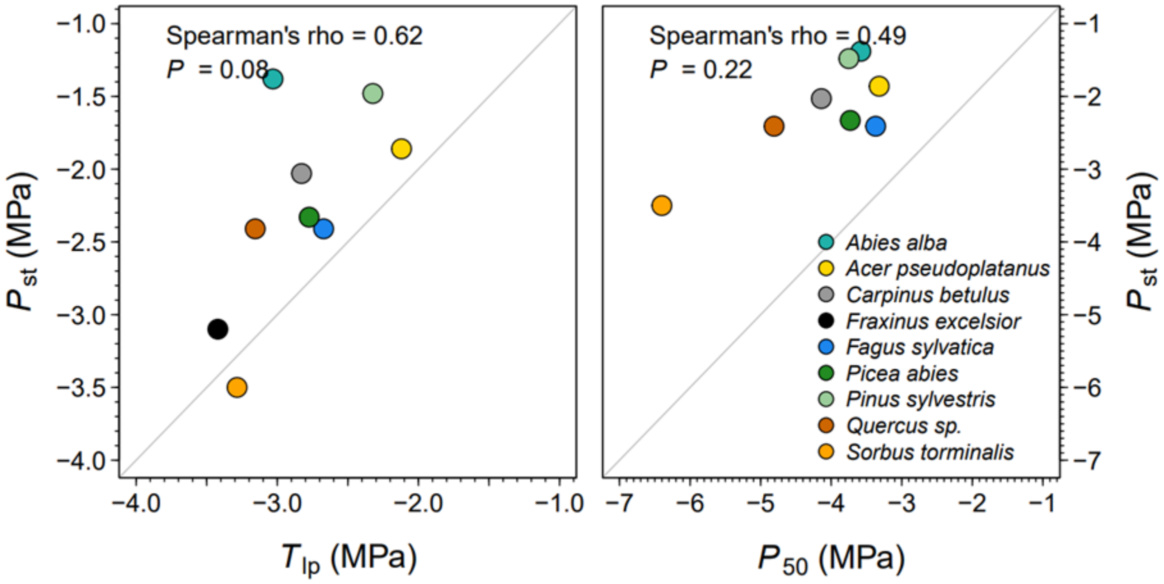

Supplementary Figure 6. Relationship between turgor loss point (T_{lp}), embolism vulnerability (P_{50}), and the midday point of stomatal closure (P_{st}). This figure illustrates the relationship between species average values of P_{50} and T_{lp} with P_{st}, as detailed in Supplementary Figure 4. Due to the presence of outliers (e.g., *Sorbus torminalis*), a Spearman's rho rank correlation test was used to assess the significance of the relationship between the dependent (P_{st}) and independent variables (P_{50} and T_{lp}).

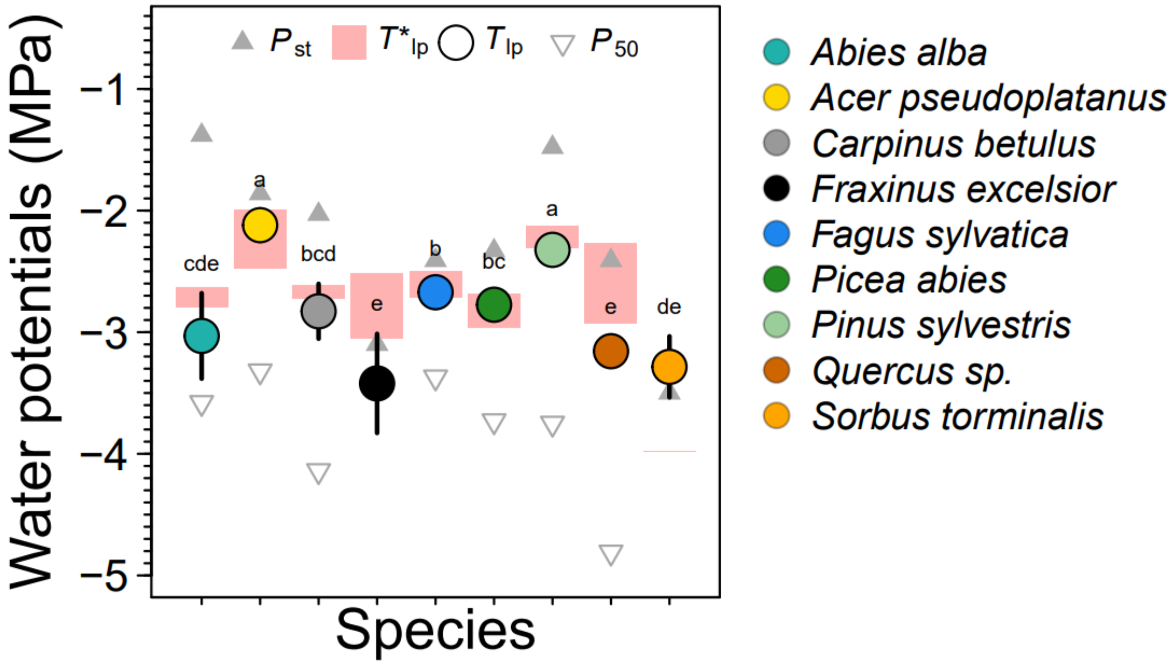

Supplementary Figure 7. Species-specific water potential threshold for the point of midday stomatal closure (P_{st}), leaf turgor loss point (T_{lp}) and embolism vulnerability (P_{50}). This figure illustrates the distance between P_{st} at midday leaf water potentials, and the measured T_{lp}. For reference the turgor loss point ranges have also been provided from literature (T^{*}_{lp}, Supplementary Table 4). The lines present the confidence intervals of the T_{lp} measurements. The water potential at which 50% of xylem is embolized (P_{50}) is taken from Supplementary Table 4. The letters indicate the statistical difference between the species-specific T_{lp} values.

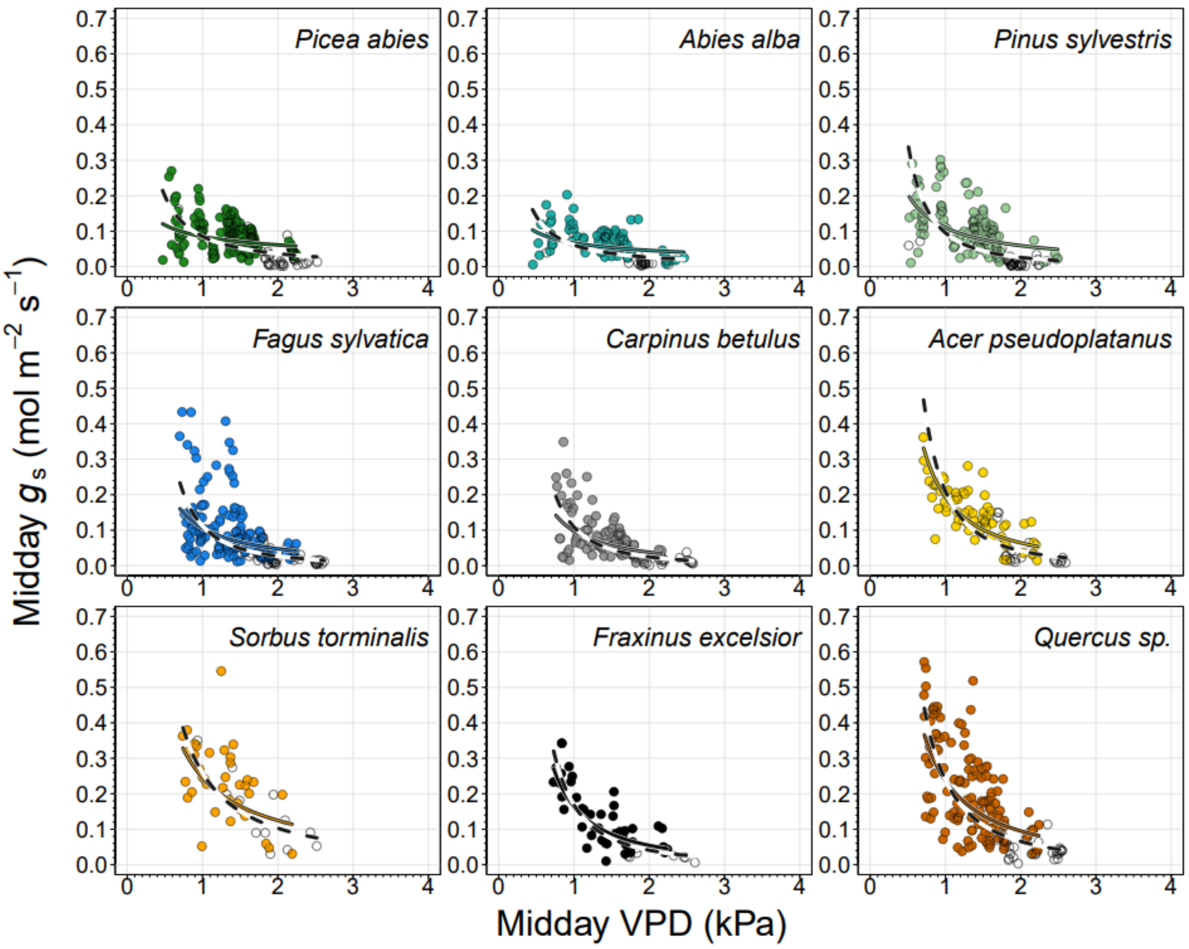

Supplementary Figure 8. Midday stomatal conductance (g_s) response to vapor pressure deficit (VPD) for the target species in Hölstein, Switzerland. Each panel shows the species-specific response of g_s and VPD. Data points include all data (open circles) and well-hydrated periods (colored circles), where pre-dawn Ψ_{leaf} values were not more negative than -1 MPa. Linear-mixed effect model fits are provided for both all data (dashed line) and well-hydrated conditions (colored line).

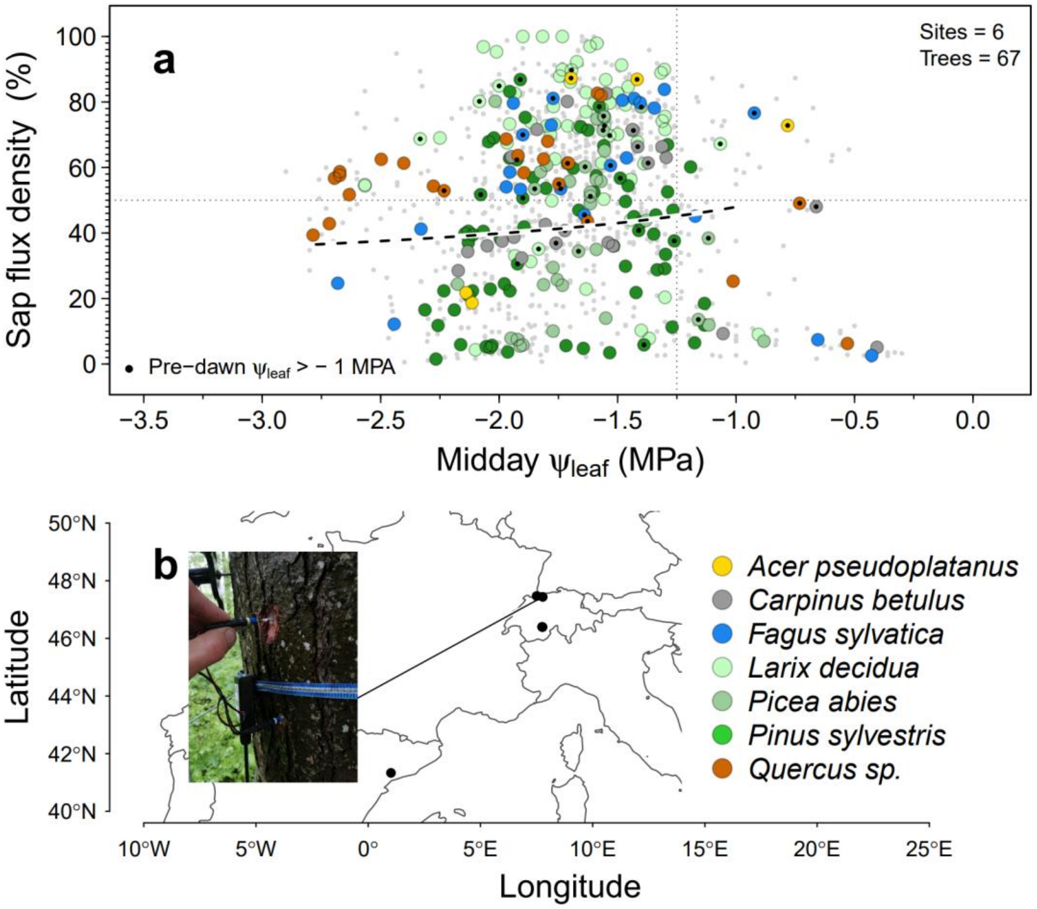

Supplementary Figure 9. Sap flow response to midday Ψ_{leaf} from European forest monitoring sites. (a) Midday Ψ_{leaf} measurements related to maximum daily sap flux density (expressed as a percentage of the tree's maximum sap flux density). Species are indicated by different colors. The line represents a non-significant log-transformed relationship between the dependent and independent variables, considering species as a random effect. The grey dots present the raw measurements, while the larger circles show the date-specific average values per site and species. Black dots illustrate data points where pre-dawn Ψ_{leaf} was not strongly negative. Dotted lines indicate the 50% value of each variable. (b) Sites included in this analysis, as presented in Supplementary Table 4. An image of a thermal dissipation method sap flow sensors is also provided.

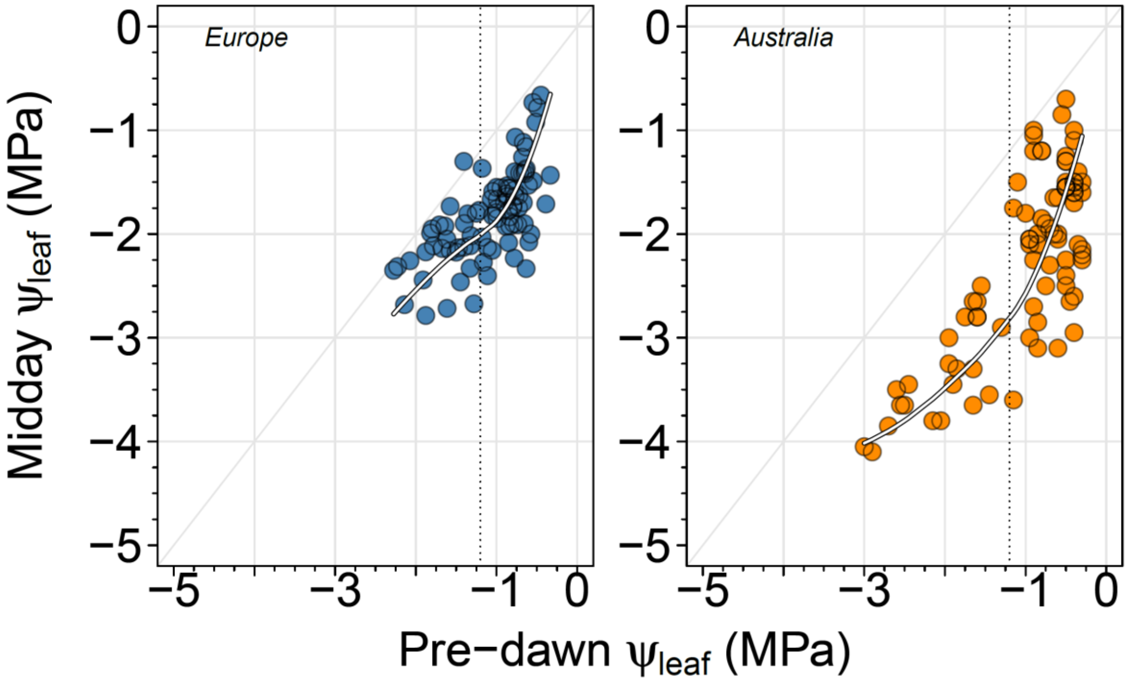

Supplementary Figure 10. Relationship between pre-dawn and midday Ψ_{leaf} for the European sap flow sites and the Australian g_s data. The European data and the Australian are presented in Fig. 3. For each dataset, raw data points of midday and pre-dawn Ψ_{leaf} are shown, with their relationship depicted using a LOESS smoother (white bold lines). The dotted line indicates the point at which pre-dawn Ψ_{leaf} is -1.2 MPa.

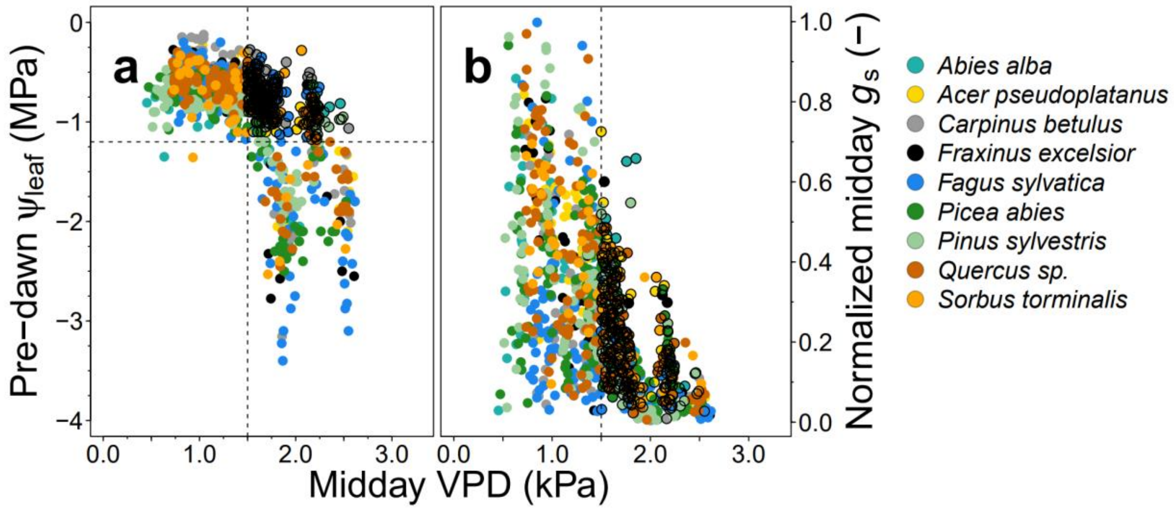

Supplementary Figure 11. Collinearity between pre-dawn leaf water potential (ψ_{leaf}) and midday vapour pressure deficit (VPD) and its impact on normalized stomatal conductance (g_s). (a) Relationship between pre-dawn ψ_{leaf} and midday VPD for each of the monitored species (in colours). The point with low pre-dawn ψ_{leaf} (> - 1.2 MPa) and high VPD (>1.5 kPa) are highlighted with black circles. (b) The response of normalized midday g_s to midday VPD for each species, with the black circles highlighting the same values as presented in (a).

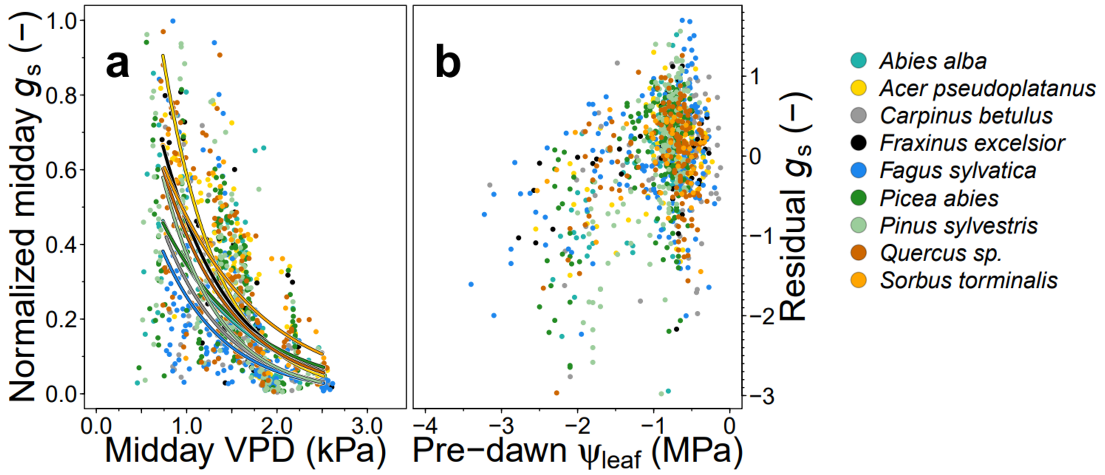

Supplementary Figure 12. Response of normalized midday stomatal conductance (g_s) against midday vapour pressure deficit (VPD) and the modelling residuals. (a) A linear mixed-effect model was fitted to explain the response of log(g_s) to log(VPD). The lines are the fits to the individual species distinguishable by colour. (b) The residuals of the model are plotted against pre-dawn leaf water potential (ψ_{leaf}).

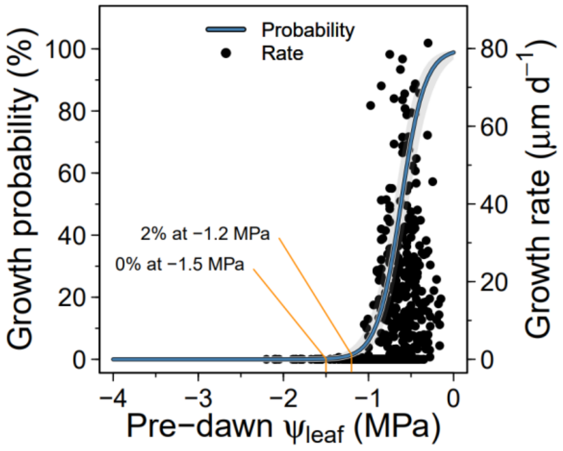

Supplementary Figure 13. Growth probability and rate responses to pre-dawn Ψ_{leaf}, obtained from daily points dendrometer readings. Zero growth rates versus non-zero growth rates were analysed to determine the growth probability using a mixed-effect model with a binomial distribution.

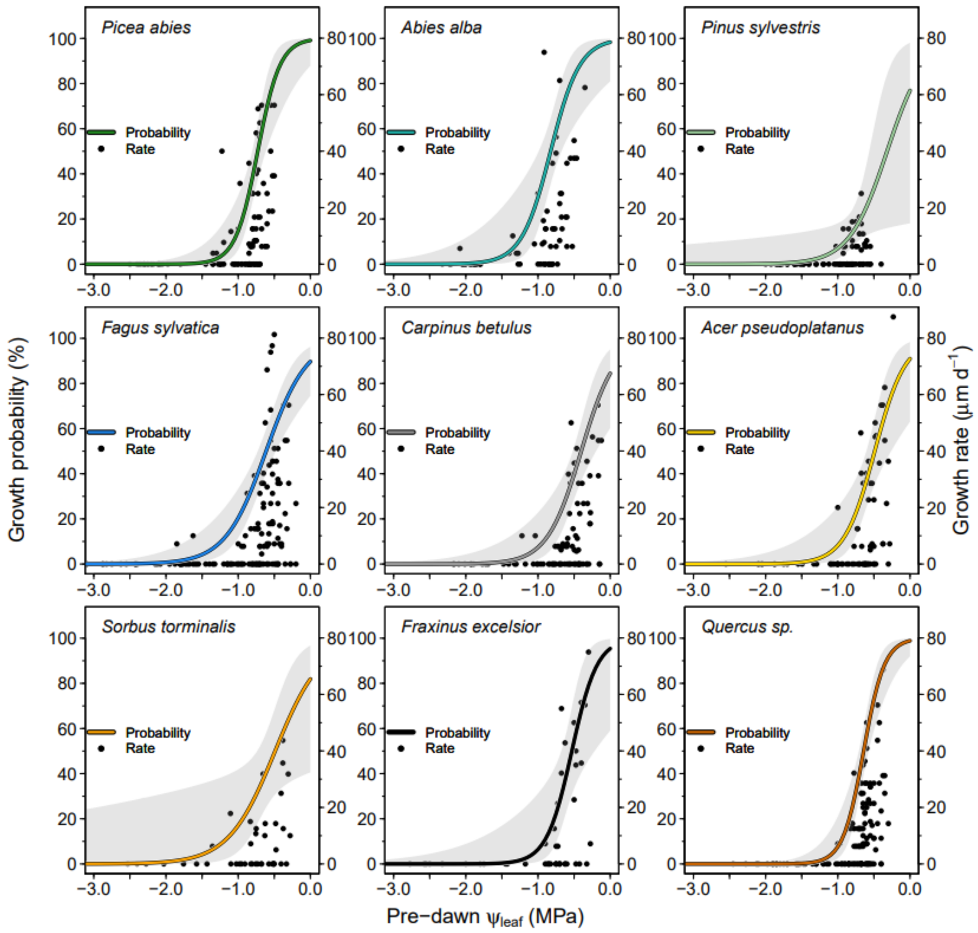

Supplementary Figure 14. Growth probability and rate responses to pre-dawn Ψ_{leaf}, obtained from weekly band dendrometer readings for each species. Zero growth rates versus non-zero growth rates were analysed to determine the growth probability using a mixed-effect model with a binomial distribution. Due to low replication in some of the species, we set low growth rate values (<10 µm d-1) to zero, to avoid the severe impact of outliers on the fit. Bear in mind that the uncertainty around these species-specific fits is high in some cases.

Supplementary Table 1. Summary of the 95 trees monitored at the Hölstein (Basel, Switzerland) study site. For each tree, the following information is provided: diameter at breast height (d_{stem}), tree height (h_{tree}), status (a=alive, d=dead), number of measurements, and monitoring years.

Species	Tree ID	d_{stem} (cm)	h_{tree} (m)	Status 2023 (a/d)	Measured (#)	Monitoring (yrs)
Abies alba (trees=9)	Aa175	18.8	18.5	a	16	2020-2022
	Aa189	22.1	20.9	a	6	2022
	Aa221	34.0	33.2	a	18	2020-2022
	Aa226	17.8	15.2	a	6	2022
	Aa239	23.0	21.9	a	6	2022
	Aa266	42.7	36.1	a	18	2020-2022
	Aa272	48.7	32.5	a	17	2020-2022
	Aa317	18.8	14.2	a	1	2020
	Aa336	44.8	32.6	a	17	2020-2022
Acer pseudoplatanus (trees=7)	Ap236	53.6	30.1	a	15	2020-2022
	Ap277	45.3	31.6	a	17	2020-2022
	Ap286	36.3	30.3	a	17	2020-2022
	Ap342	42.9	32.0	a	17	2020-2022
	Ap377	33.4	26.8	a	14	2020-2022
	Ap559	59.6	32.1	a	5	2022
	Ap605	35.6	25.7	a	5	2022
Carpinus betulus (trees=9)	Cb172	32.9	25.3	a	17	2020-2022
	Cb235	27.2	23.3	a	5	2022
	Cb244	22.0	14.4	a	15	2020-2022
	Cb268	23.0	22.2	a	17	2020-2022
	Cb287	19.9	18.6	a	13	2020-2022
	Cb307	25.5	22.9	a	11	2020-2022
	Cb440	36.6	30.6	d (03-2021)	6	2020
	Cb469	33.1	23.7	a	17	2020-2022
	Cb511	33.6	22.6	a	13	2020-2022
Fraxinus excelsior (trees=4)	Fe82	38.2	29.5	a	5	2022
	Fe242	45.0	33.3	a	17	2020-2022
	Fe243	35.7	32.3	a	15	2020-2022
	Fe442	36.5	28.9	a	16	2020-2022
Fagus sylvatica (trees=19)	Fs113	41.8	29.3	a	5	2022
	Fs144	41.6	32.3	a	17	2020-2022
	Fs203	46.1	28.1	a	4	2022
	Fs206	44.6	29.4	a	9	2020-2021
	Fs220	43.5	29.0	a	17	2020-2022
	Fs224	45.4	29.8	a	1	2021
	Fs249	61.7	29.7	a	5	2022
	Fs270	39.4	29.4	a	17	2020-2022
	Fs279	28.8	27.9	a	16	2020-2022
	Fs303	51.6	32.0	a	14	2020-2022
	Fs319	61.5	30.2	a	1	2020
	Fs353	63.1	29.8	a	12	2020-2021
	Fs378	33.7	30.3	a	5	2022
	Fs379	33.3	31.6	a	12	2020-2021
	Fs397	33.1	28.0	a	12	2020-2021
	Fs407	52.4	31.7	a	1	2020

	Fs453	41.0	27.1	a	5	2022
	Fs525	61.3	29.9	a	17	2020-2022
	Fs553	58.9	33.1	a	4	2022
Picea abies (trees=17)	Pa140	23.4	22.1	a	6	2022
	Pa185	35.1	29.8	a	6	2022
	Pa231	49.6	34.8	a	11	2020-2021
	Pa278	49.4	33.7	a	17	2020-2022
	Pa322	37.4	32.0	a	6	2022
	Pa325	40.4	31.6	a	12	2020-2021
	Pa328	43.1	33.6	a	11	2020-2021
	Pa339	34.8	33.7	a	6	2022
	Pa383	42.7	33.6	a	16	2020-2022
	Pa390	60.2	29.2	a	11	2020-2021
	Pa401	50.9	32.4	a	17	2020-2022
	Pa444	36.4	30.2	a	6	2022
	Pa467	64.4	33.0	a	16	2020-2022
	Pa470	55.7	34.2	a	11	2020-2021
	Pa494	42.8	28.7	a	6	2022
	Pa502	59.2	35.8	a	16	2020-2021
	Pa507	58.7	33.7	a	17	2020-2021
Pinus sylvestris (trees=13)	Ps109	46.9	33.7	a	8	2021-2022
	Ps132	57.7	30.7	a	6	2022
	Ps139	54.7	30.9	a	16	2020-2022
	Ps169	59.4	34.5	a	17	2020-2022
	Ps173	50.3	35.0	a	6	2022
	Ps181	49.6	29.3	a	6	2022
	Ps190	48.7	31.8	a	6	2022
	Ps217	47.9	35.3	a	18	2020-2022
	Ps223	42.8	30.6	a	12	2020-2021
	Ps245	46.3	33.6	a	18	2020-2022
	Ps247	55.4	27.7	a	5	2022
	Ps255	35.0	29.3	a	8	2021-2022
	Ps302	48.9	33.0	a	17	2020-2022
Quercus sp. petraea/robur (trees=14)	Qs167	67.6	30.1	a	17	2020-2022
	Qs214	53.0	28.3	a	5	2022
	Qs216	34.1	30.7	a	5	2022
	Qs233	48.8	29.8	a	16	2020-2022
	Qs257	48.5	31.9	a	17	2020-2022
	Qs259	61.2	32.3	a	17	2020-2022
	Qs274	57.0	32.2	a	17	2020-2022
	Qs296	78.8	33.3	a	8	2021-2022
	Qs318	39.2	29.7	a	3	2021
	Qs343	74.0	33.3	a	5	2022
	Qs389	58.7	30.7	a	17	2020-2022
	Qs398	66.2	32.5	a	17	2020-2022
	Qs433	60.3	30.0	a	1	2021
	Qs528	72.3	29.5	a	17	2020-2022
Sorbus torminalis (trees=3)	St79	24.4	19.3	a	16	2020-2022
	St300	40.6	26.9	a	17	2020-2022
	St465	35.6	22.4	a	16	2020-2022

Supplementary Table 2. Tree species description and data overview. The mean values are provided, with data ranges indicated in brackets.

Tree species	Number of trees	Number of observations	Diameter at breast height (cm)	Tree height (m)
Abies alba	9	105	33 (21-51)	25 (14-36)
Acer pseudoplatanus	7	87	44 (33-60)	30 (26-32)
Carpinus betulus	9	112	28 (20-37)	23 (14-31)
Fraxinus excelsior	4	53	39 (36-45)	31 (29-33)
Fagus sylvatica	19	164	46 (29-62)	30 (27-33)
Picea abies	17	184	46 (23-64)	32 (22-36)
Pinus sylvestris	13	143	50 (35-59)	32 (28-35)
Quercus sp.	14	162	56 (33-79)	31 (28-33)
Sorbus torminalis	3	49	34 (24-41)	23 (19-27)

Supplementary Table 3. Summary statistics of the leaf water potential (Ψ_{leaf}) and stomatal conductance (g_s). For each tree, the maximum (Max.) and minimum (Min.) values of the Ψ_{leaf} and g_s time-series were calculated. The species mean and standard deviation are provided. Only trees with at least five sampling dates were included to ensure that seasonal dynamics are visible (Supplementary Table 1).

Tree species	Min. pre-dawn Ψ_{leaf} (MPa)	Max. pre-dawn Ψ_{leaf} (MPa)	Min. midday Ψ_{leaf} (MPa)	Max. midday Ψ_{leaf} (MPa)	Max. g_s (mol m^{-2} s^{-1})	Min. g_s (mol m^{-2} s^{-1})
Abies alba	-2.051±0.18	-0.545±0.13	-2.301±0.13	-1.073±0.10	0.126±0.05	0.007±0.003
Acer pseudoplatanus	-1.904±0.41	-0.361±0.10	-2.332±0.30	-0.793±0.14	0.251±0.07	0.012±0.004
Carpinus betulus	-1.952±0.61	-0.224±0.11	-2.402±0.51	-0.580±0.16	0.193±0.09	0.008±0.005
Fraxinus excelsior	-2.425±0.43	-0.369±0.10	-3.244±0.16	-0.669±0.10	0.259±0.08	0.015±0.01
Fagus sylvatica	-2.325±0.68	-0.386±0.12	-2.948±0.49	-0.945±0.30	0.263±0.12	0.011±0.01
Picea abies	-1.966±0.49	-0.602±0.09	-2.324±0.18	-1.127±0.19	0.167±0.05	0.013±0.01
Pinus sylvestris	-1.679±0.23	-0.596±0.09	-1.961±0.13	-1.115±0.17	0.196±0.08	0.008±0.01
Quercus sp.	-1.877±0.32	-0.438±0.10	-2.825±0.13	-0.742±0.11	0.395±0.14	0.027±0.01
Sorbus torminalis	-2.230±0.41	-0.321±0.05	-4.130±0.12	-0.988±0.15	0.412±0.12	0.057±0.03

Supplementary Table 4. Branch and leaf hydraulic traits. Tree species specific turgor loss points (T_{lp}) from the target trees and literature (T^*_{lp}) and embolism vulnerability (P_{50}) according site-specific measurements. Additionally, the midday point of stomatal closure (P_{st}), as determined in Supplementary Figure 4 is provided. The confidence interval (CI) for the T_{lp} is provided.

Tree species	T_{lp} (MPa) [CI]	T^*_{lp} min/max (MPa)	P_{50} (MPa)	Sources	P_{st} (MPa)
Abies alba	-3.03 [-3.38, -2.68]	-2.63/-2.79	-3.58	Kahmen et al. 2022[26]	-1.380
Acer pseudoplatanus	-2.12 [-2.25, -1.99]	-1.99/-2.47	-3.32	Kunert & Tomoskova 2020[27]	-1.860
Carpinus betulus	-2.83 [-3.05, -2.60]	-2.61/-2.72	-4.14		-2.027
Fraxinus excelsior	-3.42 [-3.83, -3.01]	-2.51/-3.05	…		-3.102
Fagus sylvatica	-2.67 [-2.79, -2.56]	-2.50/-2.72	-3.37		-2.407
Picea abies	-2.77 [-2.88, -2.67]	-2.68/-2.96	-3.73		-2.333
Pinus sylvestris	-2.32 [-2.41, -2.23]	-2.13/-2.31	-3.75		-1.481
Quercus sp.	-3.16 [-3.28, - 3.03]	-2.27/-2.93	-4.81		-2.411
Sorbus torminalis	-3.28 [-3.54, -3.03]	-3.98/…	-6.40	*This study in accordance with the approach presented in Bartlett *et al.* 2012[28]	-3.505

Supplementary Table 5. Linear mixed-effect modelling output. Analysis of variance table for two linear mixed-effect models for midday g_s as a function of midday vapor pressure deficit (VPD), pre-dawn or midday leaf water potential (Ψ_{leaf}), and tree species.

Model	Variable	No. d.f	Den d.f.	*F*-value	*p*-value	Marginal R^2	Conditional R^2
Pre-dawn Ψ_{leaf}	Ψ_{leaf}	1	1016	427.78	<0.0001	0.68	0.70
	Species	8	318	14.19	<0.0001		
	Log(VPD)	1	1000	201.78	<0.0001		
	Ψ_{leaf} x Species	8	1020	16.30	<0.0001		
	Log(VPD) x Species	8	1003	4.93	<0.0001		
Midday Ψ_{leaf}	Ψ_{leaf}	1	1041	123.03	<0.0001	0.55	0.59
	Species	8	849	4.55	<0.0001		
	Log(VPD)	1	1015	173.98	<0.0001		
	Ψ_{leaf} x Species	8	1030	9.55	<0.0001		
	Log(VPD) x Species	8	1019	5.10	<0.0001		

Supplementary Table 6. Site description of included studies. Description of sites where Granier-type thermal dissipation probe data or stomatal conductance (g_s) measurements were collected in combination with pre-dawn and midday leaf water potential (Ψ_{leaf}) measurements. Sites where g_s measurements were collected in Australia are indicated with an asterisk (*).

Site	Elevation (m a.s.l.)	Longitude (°E)	Latitude (°N)	Species (trees)	Source
Hölstein	550	7.776	47.439	*Abies alba* (4); *Acer pseudoplatanus* (3); *Carpinus betulus* (4); *Fagus sylvatica* (6); *Quercus* sp. (5); *Picea abies* (5); *Pinus sylvestris* (6)	This study
Hofstetten	550	7.502	47.469	Picea abies (4); *Pinus sylvestris* (4); *Larix decidua* (4); *Quercus petraea* (4); *Fagus sylvatica* (4); *Carpinus betulus* (4)	Peters *et al.* 2023[5]
Lötschental	1300 (dry)	7.764	46.393	*Picea abies* (3); *Larix decidua* (1)	Peters *et al.* 2021[7]
	1300 (wet)	7.764	46.393	*Picea abies* (3); *Larix decidua* (3)	
	2200	7.743	46.340	*Larix decidua* (3)	
Tillar	1018	1.014	41.333	*Pinus sylvestris* (5)	Poyatos *et al.* 2013[8]
Darwin*	1705	130.88	-12.43	*Eucalyptus miniate* (3); *Eucalyptus tetrodonta* (3); *Acacia auriculiformis* (3)	Kahmen *et al.* 2013[9]
Katherine*	1119	132.36	-14.47	*Corymbia foelscheana* (3); *Erythrophleum chlorostachys* (3); *Eucalyptus tectifica* (3)	
Elliott*	596	133.51	-17.50	*Corymbia terminalis* (3); *Eucalyptus pruinosa* (3); *Acacia colei* (3)	
Tennant creek*	455	134.16	-19.65	*Eucalyptus leucophloia* (3); *Eucalyptus pruinose* (3); *Acacia cowleana* (3)	
Alice springs*	278	133.83	-23.70	*Corymbia aparrerinja* (3); *Corymbia terminalis* (3); *Acacia kempiana* (3)	

References

1. Babst, F. *et al.* Modeling Ambitions Outpace Observations of Forest Carbon Allocation. *Trends Plant Sci* **26**, 210–219 (2021).

2. Zahnd, C., Arend, M., Kahmen, A. & Hoch, G. Microclimatic gradients cause phenological variations within temperate tree canopies in autumn but not in spring. *Agric For Meteorol* **331**, 109340 (2023).

3. Duursma, R. A. Plantecophys-An R Package for Analysing and Modelling Leaf Gas Exchange Data. *PLoS One* **10**, e0143346 (2015).

4. Brügger, R. & Vassella, A. *Pflanzen Im Wandel Der Jahreszeiten. Anleitung Für Phanologische Beobachtungen*. (GEOGRAPHICA BERNENSIA, Bern, 2003). doi:10.4480/GB2018.N02.

5. Peters, R. L. *et al.* Daytime stomatal regulation in mature temperate trees prioritizes stem rehydration at night. *New Phytologist* **239**, 553–546 (2023).

6. Peters, R. L. *et al.* Contrasting stomatal sensitivity to temperature and soil drought in mature alpine conifers. *Plant Cell Environ* **42**, 1674–1689 (2019).

7. Peters, R. L. *et al.* Turgor – a limiting factor for radial growth in mature conifers along an elevational gradient. *New Phytologist* **229**, 213–229 (2021).

8. Poyatos, R., Aguadé, D., Galiano, L., Mencuccini, M. & Martínez-Vilalta, J. Drought-induced defoliation and long periods of near-zero gas exchange play a key role in accentuating metabolic decline of Scots pine. *New Phytologist* **200**, 388–401 (2013).

9. Kahmen, A. *et al.* Leaf water deuterium enrichment shapes leaf wax n-alkane δD values of angiosperm plants II: Observational evidence and global implications. *Geochim Cosmochim Acta* **111**, 50–63 (2013).

10. Granier, A. Une nouvelle méthode pour la mesure du flux de sève brute dans le tronc des arbres. *Ann. For. Sci.* **42**, 193–200 (1985).

11. Peters, R. L. *et al.* Assimilate, process and analyse thermal dissipation sap flow data using the TREX r package. *Methods Ecol Evol* **12**, 342–350 (2021).

12. Hurley, A. G., Peters, R. L., Pappas, C., Steger, D. N. & Heinrich, I. Addressing the need for interactive, efficient, and reproducible data processing in ecology with the datacleanr R package. *PLoS One* **17**, e0268426- (2022).

13. Pinheiro, J., Bates, D. & R Core Team. nlme: Linear and Nonlinear Mixed Effects Models. Preprint at https://cran.r-project.org/package=nlme (2023).

14. Bates, D., Mächler, M., Bolker, B. & Walker, S. Fitting Linear Mixed-Effects Models Using lme4. *J Stat Softw* **67**, 1–48 (2015).

15. Lenth, R., Singmann, H., Love, J., Buerkner, P. & Herve, M. Package "Emmeans". Preprint at http://cran.r-project.org/package=emmeans (2018).

16. Grossiord, C. *et al.* Plant responses to rising vapor pressure deficit. *New Phytologist* **226**, 1550–1566 (2020).

17. Zuur, A. F., Ieno, E. N. & Elphick, C. S. A protocol for data exploration to avoid common statistical problems. *Methods Ecol Evol* **1**, 3–14 (2010).

18. Arend, M. *et al.* Rapid hydraulic collapse as cause of drought-induced mortality in conifers. *Proceedings of the National Academy of Sciences* **118**, e2025251118 (2021).

19. Duursma, R. A. *et al.* On the minimum leaf conductance: its role in models of plant water use, and ecological and environmental controls. *New Phytologist* **221**, 693–705 (2019).

20. Knipfer, T. *et al.* Predicting Stomatal Closure and Turgor Loss in Woody Plants Using Predawn and Midday Water Potential. *Plant Physiol* **184**, 881–894 (2020).

21. Wood, S. N. *Generalized Additive Models: An Introduction with R*. (Chapman and Hall/CRC, New York, NY, 2017). doi: https://doi.org/10.1201/9781315370279.

22. Xu, G.-Q., Farrell, C. & Arndt, S. K. Climate of origin has no influence on drought adaptive traits and the drought responses of a widely distributed polymorphic shrub. *Tree Physiol* **42**, 86–98 (2022).

23. Zweifel, R., Haeni, M., Buchmann, N. & Eugster, W. Are trees able to grow in periods of stem shrinkage? *New Phytologist* **211**, 839–849 (2016).

24. Schulte, P.J., & Hinckley, T.M. A Comparison of Pressure-Volume Curve Data-Analysis Techniques. *Journal of Experimental Botany* **36**, 1590–1602 (1985).

25. Tyree, M.T., & Jarvis, P.G. Water in tissues and cells. In: Lange OL, Nobel PS, Osmond CB, Ziegler H (eds) Encyclopedia of Plant Physiology, Vol. 12B. SpringerVerlag, Berlin and New York, pp 35–77 (1982).

26. Kahmen, A. *et al.* Root water uptake depth determines the hydraulic vulnerability of temperate European tree species during the extreme 2018 drought. *Plant Biol* **24**, 1224–1239 (2022).

27. Kunert, N. & Tomaskova, I. Leaf turgor loss point at full hydration for 41 native and introduced tree and shrub species from Central Europe. *Journal of Plant Ecology* **13**, 754–756 (2020).

28. Bartlett, M. K., Scoffoni, C. & Sack, L. The determinants of leaf turgor loss point and prediction of drought tolerance of species and biomes: a global meta-analysis. *Ecol Lett* **15**, 393–405 (2012).
