# Supplementary material for: Uniform regulation of stomatal closure across temperate tree species to sustain nocturnal turgor and growth
Source: Nat Plants. 2025 Apr 3;11(4):725–30. doi: 10.1038/s41477-025-01957-3 (PMC12014480; doi:10.1038/s41477-025-01957-3)
Supplement: Supplementary file 2 — Reporting Summary [file 41477_2025_1957_MOESM2_ESM.pdf]

nature portfolio

Corresponding author(s): RICHARD L. PETERS
Last updated by author(s): Feb 12, 2025

Reporting Summary

Nature Portfolio wishes to improve the reproducibility of the work that we publish. This form provides structure for consistency and transparency in reporting. For further information on Nature Portfolio policies, see our Editorial Policies and the Editorial Policy Checklist.

Statistics

For all statistical analyses, confirm that the following items are present in the figure legend, table legend, main text, or Methods section.

n/a	Confirmed	
☐	☒	The exact sample size (*n*) for each experimental group/condition, given as a discrete number and unit of measurement
☐	☒	A statement on whether measurements were taken from distinct samples or whether the same sample was measured repeatedly
☐	☒	The statistical test(s) used AND whether they are one- or two-sided<br>*Only common tests should be described solely by name; describe more complex techniques in the Methods section.*
☐	☒	A description of all covariates tested
☐	☒	A description of any assumptions or corrections, such as tests of normality and adjustment for multiple comparisons
☐	☒	A full description of the statistical parameters including central tendency (e.g. means) or other basic estimates (e.g. regression coefficient) AND variation (e.g. standard deviation) or associated estimates of uncertainty (e.g. confidence intervals)
☐	☒	For null hypothesis testing, the test statistic (e.g. *F*, *t*, *r*) with confidence intervals, effect sizes, degrees of freedom and *P* value noted<br>*Give P values as exact values whenever suitable.*
☒	☐	For Bayesian analysis, information on the choice of priors and Markov chain Monte Carlo settings
☒	☐	For hierarchical and complex designs, identification of the appropriate level for tests and full reporting of outcomes
☒	☐	Estimates of effect sizes (e.g. Cohen's *d*, Pearson's *r*), indicating how they were calculated

Our web collection on statistics for biologists contains articles on many of the points above.

Software and code

Policy information about availability of computer code

Data collection	The stomatal conductance measurements were obtained with the program provided on the LI-6800 Portable Photosynthesis System (LI-COR Biosciences GmbH, Bad Homburg, Germany). In Australia, stomatal conductance measurements were obtained by using the software on the LI-COR 1600 Steady-State-Porometer (LiCor Inc., Lincoln, Nebraska, USA). The needle area was then extracted using a dedicated digital image analysis tool (github.com/dabasler/LeafAreaExtraction). At all sites, measurements of Ψleaf were performed by using a Scholander-type pressure chamber (PMS Instrument Company, Albany, Oregon, USA).
Data analysis	All data analyses were performed within the R software environment (Version: 4.2.2, R Core Team 2022). Openly available R packages were used for the analyses, including "datacleanr" (V: 1.0.3), "nlme" (V: 3.1-165), "lme4" (V: 1.1-35.5), "emmeans" (V: 1.10.3), "mgcv" (V: 1.9-1), "plantecophys" (V: 1.4-6), and "TREX" (V: 1.0.2, Location: https://github.com/the-Hull/TREX).

For manuscripts utilizing custom algorithms or software that are central to the research but not yet described in published literature, software must be made available to editors and reviewers. We strongly encourage code deposition in a community repository (e.g. GitHub). See the Nature Portfolio guidelines for submitting code & software for further information.

Data

Policy information about availability of data

All manuscripts must include a data availability statement. This statement should provide the following information, where applicable:

- Accession codes, unique identifiers, or web links for publicly available datasets
- A description of any restrictions on data availability
- For clinical datasets or third party data, please ensure that the statement adheres to our policy

All data are available in the main text, supplementary materials, and supplementary data files supplied with the initial submission. The gas exchange, sap flow, leaf water potential, and growth data used in this study are available in the Zenodo repository under the accession code https://doi.org/10.5281/zenodo.14852038.

Research involving human participants, their data, or biological material

Policy information about studies with human participants or human data. See also policy information about sex, gender (identity/presentation), and sexual orientation and race, ethnicity and racism.

Reporting on sex and gender	This information was not collected in this study, as it was not relevant to the main research question focused on tree physiology.
Reporting on race, ethnicity, or other socially relevant groupings	This information was not collected in this study, as it was not relevant to the main research question focused on tree physiology.
Population characteristics	This information was not collected in this study, as it was not relevant to the main research question focused on tree physiology.
Recruitment	No recruitment was needed for this study, as it did not involve human subjects.
Ethics oversight	No approval was needed for this study, as it did not involve human subjects.

Note that full information on the approval of the study protocol must also be provided in the manuscript.

Field-specific reporting

Please select the one below that is the best fit for your research. If you are not sure, read the appropriate sections before making your selection.

☐ Life sciences ☐ Behavioural & social sciences ☒ Ecological, evolutionary & environmental sciences

For a reference copy of the document with all sections, see nature.com/documents/nr-reporting-summary-flat.pdf

Ecological, evolutionary & environmental sciences study design

All studies must disclose on these points even when the disclosure is negative.

Study description	We collected unique empirical evidence to characterize the water status conditions under which mature tall trees (approximately 20-35 m in height) of various species reduce stomatal conductance (gs), considering diel leaf water potential (Ψleaf) dynamics and growth. Over three years of intensive canopy and stem monitoring of 95 trees from 9 common temperate tree species, we made observations across a wide range of environmental conditions. Using these data, we tested whether stomatal closure occurs under more uniform pre-dawn or midday Ψleaf conditions.
Research sample	For this study, we measured leaf-level stomatal conductance (gs) from sun-exposed branches in the upper canopy of trees. Concurrently, we measured leaf water potential (Ψleaf) from canopy branches. Moreover, (bi)weekly observations of stem growth at breast height were collected (using manual band dendrometers). We included broadleaved species such as Fagus sylvatica L., Acer pseudoplatanus L., Fraxinus excelsior L., Carpinus betulus L., and Sorbus torminalis Crantz, as well as conifers Picea abies Karst., Abies alba Mill., and Pinus sylvestris L. Additionally, Quercus trees, which were hybrids of Quercus petraea Liebl. and Quercus robur L. to varying degrees, were treated as a single species for this study.
Sampling strategy	We used a 50 m tall canopy crane at the Hölstein research site in Switzerland, with a 62.5 m long jib, to monitor gs and leaf water potentials (Ψleaf) in the crowns of over 95 mature trees from 9 common European species (>1000 measurements). During 35 sampling dates from 2020 to 2022, we measured concurrent midday (12:00 – 14:00 CET) gs and Ψleaf from 95 individual trees, as well as pre-dawn (04:00 – 06:00 CET) Ψleaf to establish tree hydration status. To validate the found patterns, data from existing studies was compiled with concurrent pre-dawn and midday Ψleaf combined with high-resolution sap flow measurements in Europe. Moreover, a unique dataset along a North Australian climate gradient was made available, featuring concurrent measurements of midday (14:00 – 15:00) gs and both midday and pre-dawn Ψleaf.
Data collection	For broadleaved trees, we selected healthy, sun-exposed leaves, whereas for conifers, we chose healthy sun-exposed second-year ramets to ensure fully developed leaves throughout the growing season. For each tree, we selected 2 to 3 small apical branches, 5-10

	cm long, with multiple healthy sun-exposed leaves or needles attached to it. Data was collected by: Richard L. Peters, Matthias Arend, Cedric Zahnd, Günter Hoch, Tobias Zhorzel, and Ansgar Kahmen. Data from published sources was provided by: Stefan Arndt, Lucas Cernusak, and Rafael Poyatos.
Timing and spatial scale	Diurnal campaigns were typically conducted from May until October for three years (2020-2022), when leaves were fully developed. Each campaign (35 in total) included pre-dawn sampling before sunrise (04:00 – 06:00 CET) and midday sampling (12:00 – 14:00 CET).
Data exclusions	Environmental monitoring data and sap flow data were inspected for outliers using the "datacleanr" package. Erroneous data points, such as drastic single outlier values, abrupt jumps, and random noise generated by sensor failure, were identified and removed. For the stomatal conductance (gs) measurements, we normalized our data by dividing each gs value by the maximum gs value recorded for the species. This maximum value was excluded from the analyses to prevent data inflation towards a value of 1. Additionally, negative gs values were excluded from the analysis, as these values result from sensor inaccuracies. We applied the zero-growth concept to the band dendrometer data before calculating growth rates to ensure that no negative growth, caused by drought-induced shrinkage, was included in the analyses. For the analyses, we only considered data from June, July, and August to avoid the inclusion of growth halt due to winter dormancy of the cambium.
Reproducibility	We tested whether pre-dawn Ψleaf is more constraining to whole-tree transpiration across multiple monitoring sites in Europe and Australia. However, the data consist solely of natural observations.
Randomization	The tallest trees within the crane's range from each of the nine species were selected as target trees, representing the primary contributors to forest stand transpiration. A similar sampling strategy was applied in the other included studies.
Blinding	Blinding was not relevant to our study because we focused on obtaining natural observations rather than applying an experimental design.

Did the study involve field work? ☒ Yes ☐ No

Field work, collection and transport

Field conditions	Diurnal campaigns were only planned on days without rain. The field conditions were ambient temperature from May until October.
Location	Measurements were conducted at the Swiss Canopy Crane II (SCCII) site in Hölstein, Switzerland (47.439 °N, 7.776 °E, 500 m a.s.l.). Moreover, measurements were considered from published sources: the Swiss Lötschental valley, Hofstetten (Switzerland), the Tillar valley within the Poblet nature reserve (Prades Mountains, northeast Spain), and a North Australian climate gradient from Darwin to Alice springs.
Access & import/export	All research infrastructure and data collection were carried out in accordance with local governmental authorities.
Disturbance	The crane was constructed in a natural forest, with all activities designed to avoid threatening biodiversity at the site.

Reporting for specific materials, systems and methods

We require information from authors about some types of materials, experimental systems and methods used in many studies. Here, indicate whether each material, system or method listed is relevant to your study. If you are not sure if a list item applies to your research, read the appropriate section before selecting a response.

Materials & experimental systems

n/a	Involved in the study	
☒	☐	Antibodies
☒	☐	Eukaryotic cell lines
☒	☐	Palaeontology and archaeology
☒	☐	Animals and other organisms
☒	☐	Clinical data
☒	☐	Dual use research of concern
☐	☒	Plants

Methods

n/a	Involved in the study	
☒	☐	ChIP-seq
☒	☐	Flow cytometry
☒	☐	MRI-based neuroimaging

Dual use research of concern

Policy information about dual use research of concern

Hazards

Could the accidental, deliberate or reckless misuse of agents or technologies generated in the work, or the application of information presented in the manuscript, pose a threat to:

No	Yes	
☒	☐	Public health
☒	☐	National security
☒	☐	Crops and/or livestock
☒	☐	Ecosystems
☒	☐	Any other significant area

Experiments of concern

Does the work involve any of these experiments of concern:

No	Yes	
☒	☐	Demonstrate how to render a vaccine ineffective
☒	☐	Confer resistance to therapeutically useful antibiotics or antiviral agents
☒	☐	Enhance the virulence of a pathogen or render a nonpathogen virulent
☒	☐	Increase transmissibility of a pathogen
☒	☐	Alter the host range of a pathogen
☒	☐	Enable evasion of diagnostic/detection modalities
☒	☐	Enable the weaponization of a biological agent or toxin
☒	☐	Any other potentially harmful combination of experiments and agents

Plants

Seed stocks	No plants were removed from their natural locations, and all observations were conducted in the field.
Novel plant genotypes	No novel plant genotypes were produced.
Authentication	No authentication procedure was required.
